# Alveolar type 2 cells marker gene *SFTPC* inhibits epithelial-to-mesenchymal transition by upregulating *SOX7* and suppressing WNT/β-catenin pathway in non-small cell lung cancer

**DOI:** 10.3389/fonc.2024.1448379

**Published:** 2024-09-13

**Authors:** Qiongyin Zhang, Ning An, Yang Liu, Ying Zhu, Wuliang Pan, Peiling Gu, Jinzhu Zhao, Qiang Pu, Wen Zhu

**Affiliations:** ^1^ State Key Laboratory of Biotherapy, West China Hospital, Sichuan University, Chengdu, Sichuan, China; ^2^ Cancer Center, Sichuan Provincial People’s Hospital, School of Medicine, University of Electronic Science and Technology of China, Chengdu, Sichuan, China; ^3^ Department of Thoracic Surgery, West China Hospital, Sichuan University, Chengdu, Sichuan, China

**Keywords:** SFTPC, NSCLC, EMT, SOX7, Wnt/β-Catenin pathway

## Abstract

**Introduction:**

Surfactant Protein C gene (*SFTPC*) is a marker gene of alveolar type 2 cells (AT2), which are the key structures of alveoli. Mutations or deletions in *SFTPC* cause idiopathic pulmonary fibrosis (IPF). Importantly, IPF is an independent risk factor for non-small cell lung cancer (NSCLC). It suggests that abnormal expression of *SFTPC* may be relevant to development of NSCLC. However, the function and mechanism of *SFTPC* in NSCLC are still poor understood until now.

**Methods:**

The expression of *SFTPC* and the relationship between *SFTPC* and prognosis of NSCLC were analyzed in TCGA database and our collected clinical NSCLC tissues. Subsequently, the function and mechanism of *SFTPC* in NSCLC were explored by RNA-sequence, qRT-PCR, Western blot, Immunohistochemical, Wound-healing, Millicell, Transwell assays and mouse tumor xenograft model.

**Results:**

*SFTPC* was dramatically downregulated in NSCLC tissues from TCGA database and 40 out of 46 collected clinical LUAD tissues compared with adjacent non-tumor tissues. Low expression of *SFTPC* was associated with poor prognosis of LUAD by TCGA database. Importantly, we confirmed that overexpression of *SFTPC* significantly inhibited Epithelial-to-Mesenchymal Transition (EMT) process of NSCLC cells by upregulating *SOX7* and then inactivating WNT/β-catenin pathway *in vitro* and *in vivo*. Particularly, we discovered that low expression of *SFTPC* was associated with EMT process and low expression of *SOX7* in NSCLC tissues.

**Conclusion:**

Our study revealed a novel mechanism of *SFTPC* in NSCLC development. Meanwhile, it also might provide a new clue for exploring the molecular mechanism about NSCLC development in patients with IPF in the future.

## 1 Introduction

Lung cancer (LC) is the leading cause of cancer death globally (1). Non-small cell lung cancer (NSCLC), which mainly includes lung adenocarcinoma (LUAD) and lung squamous cell carcinoma (LUSC), is the main subtype of LC, accounting for about 85% of all subtypes (2). Unfortunately, about 70% of NSCLC patients were diagnosed at advanced stage, the 5-year overall survival (OS) rate was only 15% (3). Therefore, it is necessary to explore new molecular mechanisms of NSCLC development. The mammalian lung is a vital organ responsible for gas exchange. The alveoli, which account for 99% of lung’s surface area, are respiratory units of the lung (4). Alveolar epithelium consists of alveolar type 1 cells (AT1) and alveolar type 2 cells (AT2) (4). AT2 cells are the key structures of alveoli. Specially, AT2 cells also serve as alveolar stem cells, they are responsible for the alveolar epithelium repair and regeneration (5). Surfactant Protein C gene (*SFTPC*) is a marker gene of AT2 cells, which encodes surfactant protein C (SP-C) (6). It had been demonstrated *SFTPC* was only significantly expressed in mature AT2 cells eventually (7).

The previous studies demonstrated that mutations or deletions in *SFTPC* could cause idiopathic pulmonary fibrosis (IPF) (8). IPF is a progressive fibrotic interstitial lung disease associated with significant morbidity, high mortality and poor prognosis (9). Epithelial-to-Mesenchymal Transition (EMT) of AT2 cells in human IPF lesion had been observed in the earlier studies (10, 11). Particularly, Luis Rodriguez et al. (12) performed RNA-sequence (RNA-seq) on the *Sftpc^C121G^* mice with spontaneous pulmonary fibrosis, and the Gene ontogeny (GO) analysis displayed that *Sftpc* mutation (*Sftpc^C121G^*) was significantly associated with cell migration. Importantly, the migration abilities of cancer cells was closely related to EMT of cells (13). Therefore, the studies above suggested that abnormal expression of *SFTPC* might induce EMT process in AT2 cells.

Strikingly, the previous studies demonstrated that abnormal AT2 cells were the origin cells of NSCLC (14, 15). Recently, some investigators revealed that AT2-like cells appeared in human early-stage LUAD tissues by single-cell sequence, and the gene expression profiles of AT2-like cells were significantly different from those of AT2 cells (16, 17). Importantly, several epidemiological researches had indicated that IPF was also one of independent risk factors for NSCLC (18, 19). Meanwhile, the latest epidemiological research in Asia showed that the incidence of NSCLC was notably increased in IPF group than that in non-IPF group, and the hazard ratios of NSCLC in IPF group was 5.89 (19). To sum up, these results above suggested that AT2 cells marker gene *SFTPC*, which was aberrant in IPF, might also play an important role in development of NSCLC.

The previous researches about abnormal expression of *SFTPC* in lung disease were mainly focused on IPF. However, only a few studies found the deletions or mutations of *SFTPC* in clinical NSCLC tissues (20–22). Recently, Antonella F.M. Dost, et al. (16) discovered *SFTPC* was markedly decreased in AT2-like cells of human early-stage LUAD tissues compared with AT2 cells. Interestingly, the latest study found that compared with AT2-like cells of LUAD *in situ*, *SFTPC* and *E-cadherin* were significantly downregulated in AT2-like cells of invasive LUAD tissues by single cell sequence and RNA fluorescence *in situ* hybridization (RNA-FISH) (17). It implied that downregulation of *SFTPC* might be related to EMT process of NSCLC cells. Several studies demonstrated that EMT process enhanced the migration abilities of NSCLC cells, which was correlation with poor prognosis of NSCLC patients (13, 23). Recently, Baile Zuo et al. (24) also discovered that low expression of *SFTPC* was markedly associated with poor prognosis of NSCLC by multiple databases. However, the function and mechanism of *SFTPC* in EMT process of NSCLC cells are still poor understood until now.

Therefore, in our present study, the function and molecular mechanism of the AT2 cells marker gene *SFTPC* in EMT process of NSCLC cells were investigated in human NSCLC cell lines, tumor xenograft models and clinical NSCLC tissues.

## 2 Materials and methods

### 2.1 NSCLC patient samples

The approvals for patient samples study were obtained from the West China Hospital of Sichuan University Biomedical Research Ethics Committee and the Medical Ethics Committee, Sichuan Academy of Medical Sciences, Sichuan Provincial People’s Hospital (Chengdu, China). The study was performed in accordance with the Declaration of Helsinki. Written informed consent was obtained from all the patient. Clinical information of all NSCLC tissues was available in **Supplementary Information** (**Supplementary Table S1**).

### 2.2 Bioinformatics

All information of bioinformatics analysis was available in **Supplementary Information**.

### 2.3 Cell lines and cell cultures

Human NSCLC cell lines A549 and H1299 were purchased from the American Type Culture Collection (ATCC, Manassas, VA, USA). Full methods were available in **Supplementary Information**.

### 2.4 RNA-sequence

RNA-seq was conducted on A549-*SFTPC* and A549-Control cells. Full methods of RNA-seq analysis were available in **Supplementary Information**.

### 2.5 Quantitative real-time PCR

qRT-PCR procedure was performed as described in the previous study (25). To analyze the relative expression levels of genes, the 2^-ΔΔCt^ method was performed on data of this experiment. All methods were available in **Supplementary Information**.

### 2.6 Western blot

Western blot procedure was performed as described in the previous study (25). All the primary antibodies were available in **Supplementary Information**.

### 2.7 Immunohistochemical staining

IHC staining was performed on the paraffin sections as described in the previous study (25). All methods and primary antibodies were available in **Supplementary Information**.

### 2.8 Wound-healing assays

6-well plastic dishes were inoculated with treated cells, then, Wound-healing was performed as described in the previous study (26). Full methods were available in the **Supplementary Information**.

### 2.9 Millcell and transwell assays

Cells were transplanted in Millicell or Transwell chambers (8μm pore size, Merck, Millipore, Switzerland), then cultured with serum-free media as described in our previous study (26). Full methods were available in **Supplementary Information**.

### 2.10 Tumor xenograft model

The animal study was approved by the West China Hospital of Sichuan University Animal Ethics Committee (Chengdu, China). The study was conducted in accordance with the local legislation and institutional requirements. Full methods were available in the **Supplementary Information**.

### 2.11 Statistical analysis

All data analyses were performed by using GraphPad Prism 9.5 software. Data were denoted as Mean ± SD. The analyses about clinical statistical were carried out using *χ2* test. Unpaired two-tailed student *t* test was utilized to determine the statistical significance of two groups. (*p* values were shown in each figure, and were presented as “*” *p*< 0.05, “**” *p*<0.01, “***” *p*<0.005 and “****” *p*<0.001.

## 3 Results

### 3.1 The expression of *SFTPC* was significantly decreased in clinical NSCLC tissues and low expression of *SFTPC* was associated with poor prognosis of LUAD

**Figure 1:**
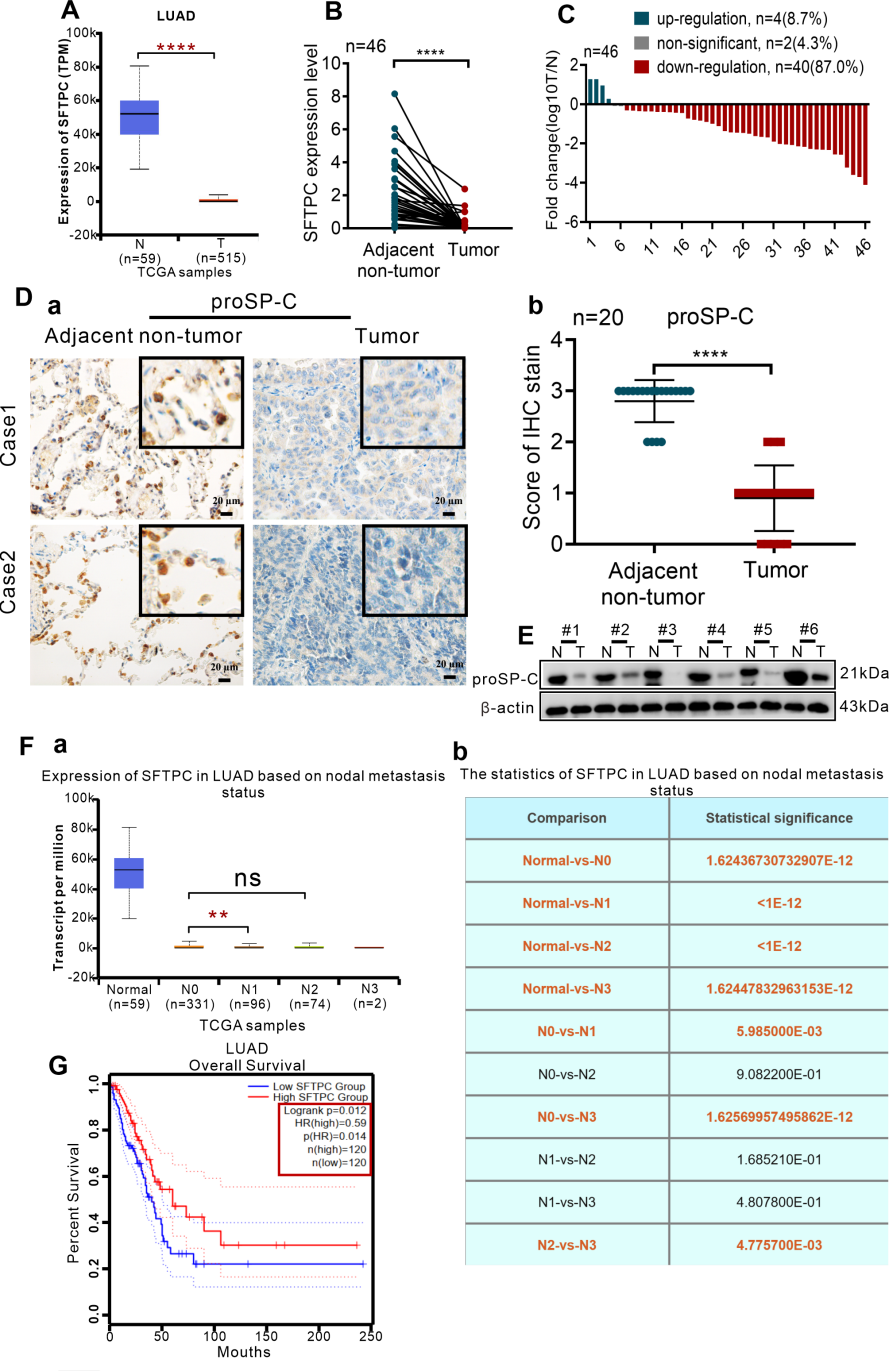
The expression of *SFTPC* was significantly decreased in clinical LUAD tissues and low expression of *SFTPC* was associated with poor prognosis of LUAD by a comprehensive analysis of TCGA database and collected clinical LUAD tissues. **(A)** The expression levels of *SFTPC* in clinical LUAD tissues and non-tumor tissues (The data were obtained from the TCGA database). T, tumor tissues. N, non-tumor tissues. **(B)** qRT-PCR analysis was carried out to assess the expression levels of *SFTPC* in collected clinical LUAD tissues and non-tumor tissues (*n*=46). β-actin was used as the internal control gene. Paired *t*-test. **(C)** The expression levels of *SFTPC* in collected clinical LUAD tissues were arranged by log10 T/N (*n*=46). T, tumor tissues. N, non-tumor tissues. **(D)** IHC staining was conducted on paraffin sections of paired clinical LUAD tissues by using primary antibody against proSP-C (*n*=20) (a). Staining without primary antibody was used as negative control. Scale bars, 20μm. The histogram was applied to quantify the results of IHC staining (b). The data were presented as the Mean ± SD, unpaired *t*-test. **(E)** Western blot assay was conducted on the protein of paired clinical LUAD tissues by using the primary antibody against proSP-C (*n*=6). **(F)** The relationship between the expression levels of *SFTPC* and lymph node metastasis of LUAD tissues (*n*=503) (a) the statistics were presented in (b) (The data were obtained from the TCGA database). **(G)** The relationship between the expression levels of *SFTPC* and the overall survival (OS) rate of LUAD patients (*n*=240). (The data were obtained from the TCGA database). *p<0.05, **p<0.01, ****p<0.001. ns, no significance.

To clarify the expression of *SFTPC* in clinical NSCLC samples, the expression of *SFTPC* was analyzed in TCGA database and 46 pairs of collected LUAD samples and their paired adjacent non-tumors. Firstly, the expression of *SFTPC* was notably downregulated in 515 LUAD cases compared with 59 adjacent non-tumors cases in TCGA database (**Figure 1A**). Then, qRT-PCR was conducted in 46 pairs of collected LUAD samples (**Supplementary Table S1**). The mRNA level of *SFTPC* was notably decreased in 40 out of 46 (40/46, 87.0%) LUAD samples compared with the matched adjacent non-tumors (**Figures 1B, C**). Meanwhile, compared with paired adjacent non-tumors, the protein level of proSP-C was observably decreased in tumors by IHC staining (**Figure 1D**) and Western blot assay (**Figure 1E**). The score criteria and outcomes for proSP-C of IHC staining were showed in **Supplementary Table S2**.

Moreover, we further investigated the correlation between *SFTPC* and prognosis of NSCLCpatients in TCGA database and 45 pairs of collected LUAD tissues. Firstly, we found that lower *SFTPC* was notably correlation with lymph node metastasis of the 503 LUAD cases in TCGA database (**Figure 1F**, **Supplementary Table S3**). Meanwhile, survival analysis was performed in 240 cases of LUAD, and indicated that lower *SFTPC* was correlated with lower OS rate of LUAD patients (**Figure 1G**). Next, the correlation between *SFTPC* and age, gender, T stage, lymph node metastasis in 45 pairs of LUAD tissues was further analyzed, respectively. Unfortunately, the low expression of *SFTPC* had no obvious relationship with age, gender, T stage and lymph node metastasis in our collected 45 pairs of LUAD samples (**Supplementary Table S4**). All of the outcomes above indicated that the expression of *SFTPC* was markedly decreased in NSCLC, and TCGA database implied low expression of *SFTPC* was correlated with poor prognosis of LUAD.

### 3.2 Overexpression of *SFTPC* inhibited migration, invasion abilities and EMT process of NSCLC cells *in vitro*

Our results proved the expression of *SFTPC* were notably decreased in clinical NSCLC tissues compared with matched adjacent non-tumors. Analogously, other researchers also found the expression of *SFTPC* was remarkably decreased in NSCLCs compared with adjacent non-tumors (24, 27, 28). Therefore, the stable *SFTPC*-overexpressing A549 and H1299 cell lines (termed A549-*SFTPC* and H1299-*SFTPC*) and control cell lines (termed A549-Control and H1299-Control) were established successfully by lentivirus infection for exploring the functions of *SFTPC* in NSCLC.

**Figure 2:**
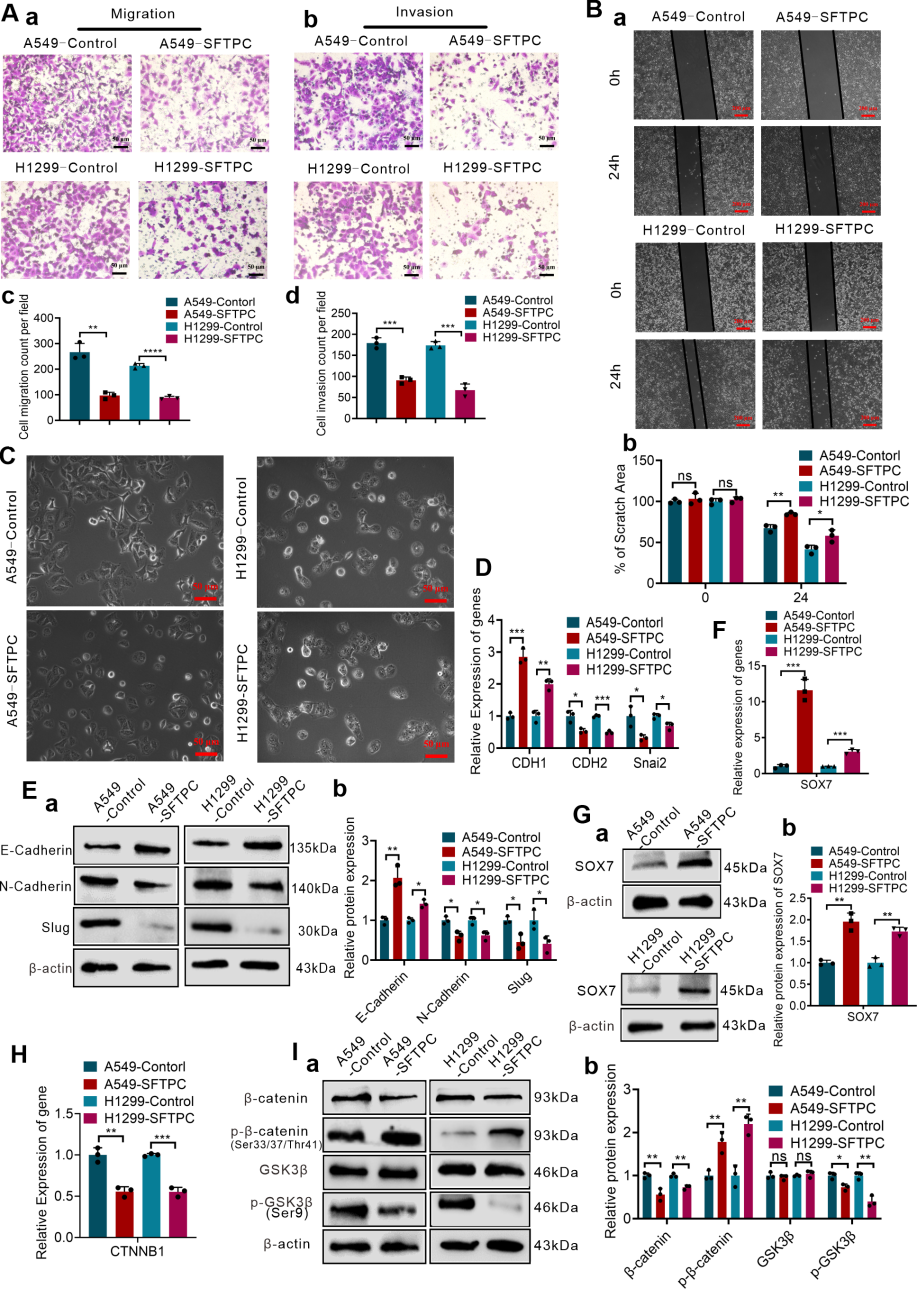
Overexpression of *SFTPC* repressed migration, invasion, EMT process, WNT/β-catenin pathway and upregulated *SOX7* in NSCLC cells. **(A)** Millicell and Transwell assays were used to detect the vertical migration and invasion abilities of indicated cells (*n*=3) (*n* represented the number of fields for counting and statistics, experiments were conducted a minimum of three times independently). The images of vertical migrated cells or invaded cells were showed in (a, b). And the histograms were applied to quantify the vertical migration or invasion abilities of indicated cells (c, d). Scale bars, 50μm. **(B)** Wound-healing assay was used to detect lateral migration ability of indicated cells (*n*=3) (*n* represented the number of pictures for statistics, experiments were conducted a minimum of three times independently). The scratch areas of indicated cells were showed in (a), Scale bars, 200μm. And the histogram was applied to quantify the lateral migration ability of cells (b). **(C)** The morphology changes of indicated cells were showed in morphological images. Scale bar, 50μm. **(D)** qRT-RCR assay was used to detect the mRNA levels of EMT markers (*CDH1*(E-cadherin), *CDH2*(N-cadherin), *Snai2*(Slug)) in indicated cells, the histogram was applied to quantify the relative mRNA levels. **(E)** Western blot assay was used to detect the protein levels of EMT markers in indicated cells by using primary antibodies against E-cadherin, N-cadherin and Slug (a), and the histogram was applied to quantify the relative protein levels (b). **(F)** qRT-RCR assay was used to detect the mRNA levels of *SOX7* in indicated cells, the histogram was applied to quantify the relative mRNA levels. **(G)** Western blot assay was used to detect the protein levels of SOX7 by using primary antibody against SOX7 in indicated cells (a), and the histogram was applied to quantify the relative protein levels (b). **(H)** qRT-RCR assay was used to detect the mRNA levels of *CTNNB1*(β-catenin) in indicated cells, the histogram was applied to quantify the relative mRNA levels. **(I)** Western blot assay was used to detect the protein levels of WNT/β-catenin pathway markers by using primary antibodies against GSK3β, p-GSK3β(Ser9), β-catenin, p-β-catenin(Ser33/Ser37/Thr41) in indicated cells (a), and the histogram was applied to quantify the relative protein levels (b). β-actin was used as the internal control gene. All data were presented as the Mean ± SD. Two groups were compared by un-paired *t*-test. *p<0.05, **p<0.01, “***”*p*<0.005 and “****”*p*<0.001. ns, no significance. All of these experiments were conducted a minimum of three times independently.

The overexpression efficiencies of *SFTPC* were verified though qRT-PCR and Western blot (**Supplementary Figures S1A–C**). Previous studies demonstrated that overexpression of *SFTPC* repressed the proliferation capacity of NSCLC cells (24, 29). In present study, we focused on the other functions of *SFTPC* in NSCLC development. Thus, to determine whether overexpression of *SFTPC* inhibited migration, invasion abilities and EMT process of NSCLC cells, the Wound-healing, Millicell and Transwell assays were performed on A549-*SFTPC*, H1299-*SFTPC* and their control cells, respectively. We uncovered that overexpression of *SFTPC* obviously repressed the migration and invasion abilities of NSCLC cells (**Figures 2A, B**). And compared to control cells, a significant cobblestone-like shape change was observed in A549-*SFTPC* cells. However, the morphology of H1299-*SFTPC* cells was not changed (**Figure 2C**). Meanwhile, compared to control cells, *CDH1*(E-cadherin) was notably increased while *CDH2*(N-cadherin) and *Snai2*(Slug) were prominently decreased in A549-*SFTPC* and H1299-*SFTPC* cells though qRT-PCR and Western blot assays (**Figures 2D, E**). Taken together, all of these changes in cell movement, cell morphology and biomarkers proved that overexpression of *SFTPC* notably repressed migration, invasion abilities and EMT process of NSCLC cells.

### 3.3 Overexpression of *SFTPC* upregulated the expression of *SOX7* and repressed WNT/β-catenin pathway *in vitro*

To gain insights into the mechanisms of stable *SFTPC*-overexpression inhibiting EMT process of NSCLC cells, RNA-seq was firstly performed in A549-*SFTPC* group and A549-Control group. A total of 953 differential expression genes (DEGs) were identified, and 331 genes were notably upregulated while 622 genes were dramatically downregulated in A549-*SFTPC* group compared with A549-Control group. (**Supplementary Figure S2A**). The distinct and consistent difference of the gene expression profile caused by stable *SFTPC*-overexpression had been shown in the hierarchical clustering heatmap (**Supplementary Figure S2B**). The GO analysis of biological process, cell components and molecular function all suggested that DEGs were associated with the characteristics of EMT process, such as cell adhesion, locomotion of cells, cell migration, extracellular matrix, and so on (**Supplementary Figure S3A**). The KEGG analysis and Reactome analysis also indicated that the DEGs were related to EMT process (**Supplementary Figures S3B, C**). The above results suggested that overexpression of *SFTPC* might inhibit EMT process of NSCLC cells.

To uncover the underlying genes of *SFTPC*, the DEGs got from RNA-seq and the two datasets “the differentially expressed genes in LUAD” and “the genes associated with *SFTPC* in LUAD” got from TCGA database were utilized to obtain the potential target genes of *SFTPC* (**Supplementary Figure S3D-a**). And in the 28 overlapping DEGs from Venn diagram, *SOX7* was found to be upregulated in A549-*SFTPC* group (**Supplementary Figure S3D-b**). Previous researches reported that *SOX7* was low expression in NSCLC tissues, which was related to lymph node metastasis and low OS rate of LUAD patients (30, 31). Meanwhile, it was reported that overexpression of *SOX7* inhibited the migration and invasion abilities of NSCLC cells (32, 33). Importantly, our qRT-PCR and Western blot analysis proved the mRNA and protein levels of SOX7 were both remarkably upregulated in A549-*SFTPC* and H1299-*SFTPC* groups compared to their control groups (**Figures 2F, G**). Thus, we demonstrated overexpression of *SFTPC* significantly upregulated the expression of *SOX7*.

And it’s worth noting that the GSEA-GO, GSEA-KEGG and GSEA-Reactome analysis suggested that overexpression of *SFTPC* was negatively correlated with WNT/β-catenin pathway (**Supplementary Figures S4A–C**). β-catenin is the core of WNT/β-catenin pathway. Here, we found that overexpression of *SFTPC* inhibited the mRNA level of *CTNNB1*(β-catenin) by qRT-PCR (**Figure 2H**). Meanwhile, compared with A549-Control and H1299-Control groups, the protein level of GSK3β was not changed, but the levels of p-GSK3β(Ser9) and β-catenin were markedly downregulated while the level of p-β-catenin (Ser33/Ser37/Thr41) was observably upregulated in A549-*SFTPC* and H1299-*SFTPC* cells by Western blot assay (**Figure 2I**). Degradation of β-catenin by phosphorylation at Thr41, Ser37 and Ser33 residues via GSK3β could lead to inactivation of the WNT/β-catenin pathway, whereas inactivation of GSK3β by phosphorylation at Ser9 residue could lead to activation of the WNT/β-catenin pathway (34). Therefore, our results indicated overexpression of *SFTPC* inactivated WNT/β-catenin pathway observably.

Taken together, we proved that overexpression of *SFTPC* upregulated the expression of *SOX7* and repressed WNT/β-catenin pathway. Interestingly, the previous studies displayed overexpression of *SOX7* suppressed WNT/β-catenin pathway, which could be mediated by interaction between SOX7 protein and β-catenin (35, 36). Meanwhile, overactivation of WNT/β-catenin was the vital activator of EMT process (13). Thus, we further hypothesized that overexpression of *SFTPC* might inhibit EMT process of NSCLC cells by upregulating *SOX7* and then repressing WNT/β-catenin pathway.

### 3.4 Overexpression of *SFTPC* inhibited migration, invasion abilities and EMT process of NSCLC cells via upregulating *SOX7 in vitro*

**Figure 3:**
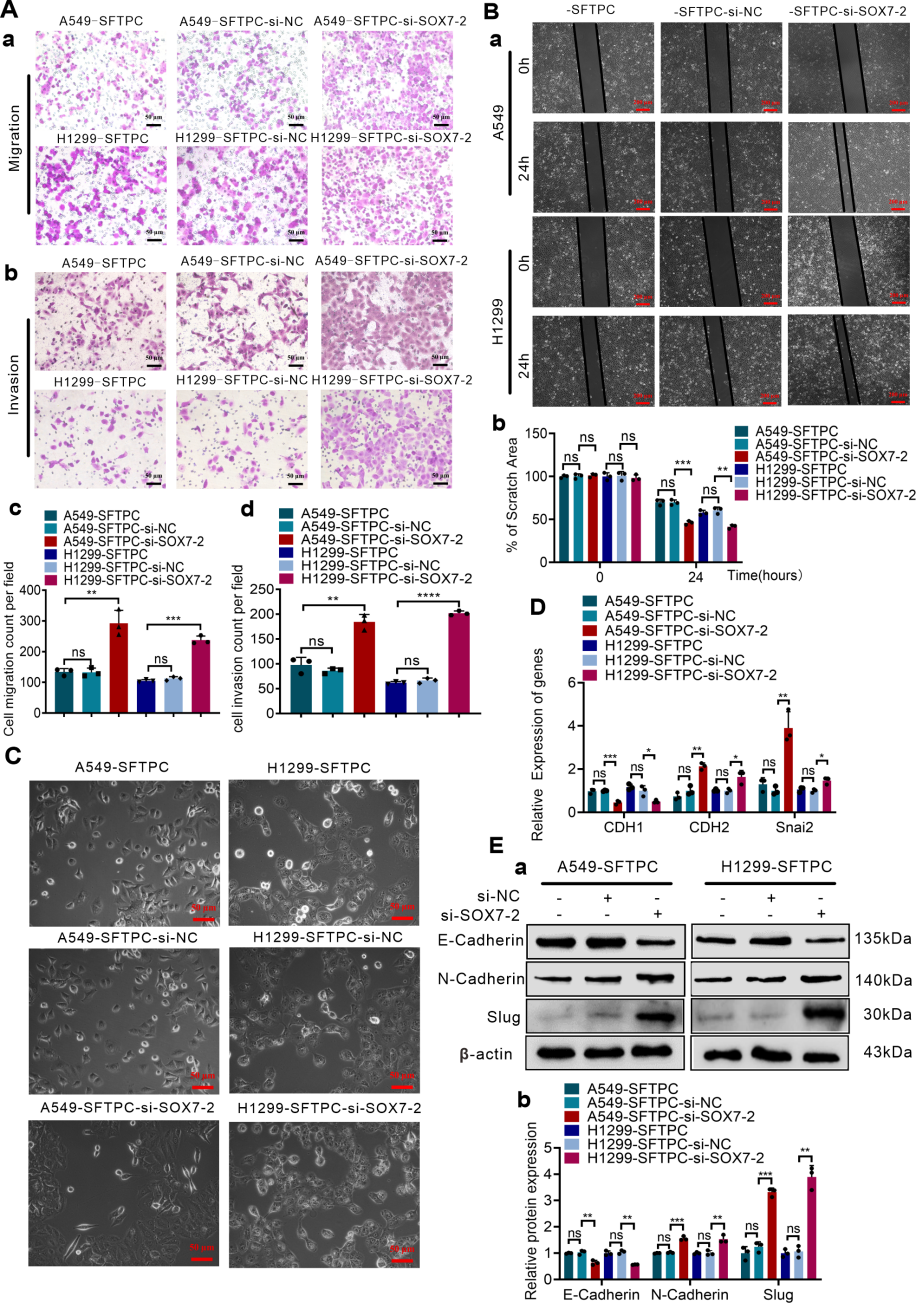
Overexpression of *SFTPC* inhibited migration, invasion abilities and EMT process of NSCLC cells via upregulation of *SOX7*. **(A)** Millcell and Transwell assay were used to detect the vertical migration and invasion abilities of the indicated cells (*n*=3) (*n* represented the number of fields for counting and statistics, experiments were conducted a minimum of three times independently). The images of vertical migrated and invaded cells were showed in (a, b). Scale bar, 50μm. The histograms were applied to quantify the vertical migration and invasion abilities of indicated cells (c, d). **(B)** Wound-healing assay was used to detect the lateral migration ability of the indicated cells (*n*=3) (*n* represented the number of pictures for statistics, experiments were conducted a minimum of three times independently). The scratch areas of indicated cells were showed in (a). Scale bar, 200μm. The histogram was applied to quantify the lateral migration ability of indicated cells (b). **(C)** The morphology changes of indicated cells were presented in morphological images. Scale bar, 50μm. **(D)** qRT-RCR assay was used to detect the mRNA levels of EMT markers [*CDH1*(E-cadherin), *CDH2*(N-cadherin), *Snai2*(Slug)]. The histogram was applied to quantify the relative mRNA levels. **(E)** Western blot assay was used to detect the protein levels of EMT markers by using primary antibodies against E-cadherin, N-cadherin and Slug (a), and the histogram was applied to quantify the relative protein levels (b). β-actin was used as the internal control gene. All the data were presented as the Mean ± SD, un-paired *t*-test. *p<0.05, **p<0.01, ***p<0.005, ****p<0.001. ns, no significance. All of these experiments were conducted a minimum of three times independently.

To explore whether overexpression of *SFTPC* inhibited migration, invasion abilities and EMT process via upregulating *SOX7* in NSCLC cells, A549-*SFTPC* and H1299-*SFTPC* cells were treatment with si-*SOX7*-2, which was the most effective sequence of si-*SOX7* (**Supplementary Figures S5A–C**). Then, the migratory and invasive capacities of A549-*SFTPC*-si-*SOX7*-2 and H1299-*SFTPC*-si-*SOX7*-2 cells were partially restored compared with A549-*SFTPC*-si-NC and H1299-*SFTPC*-si-NC cells (**Figures 3A, B**). Meanwhile, a typical spindle-like shape change was observed in A549-*SFTPC*-si-*SOX7*-2 cells compared to A549-*SFTPC*-si-NC cells, but the morphology of H1299-*SFTPC*-si-*SOX7*-2 cells was not changed compared to H1299-*SFTPC*-si-NC cells (**Figure 3C**). And *CDH1*(E-cadherin) was notably decreased while *CDH2*(N-cadherin) and *Snai2*(Slug) were markedly increased in A549-*SFTPC*-si-*SOX7*-2 and H1299-*SFTPC*-si-*SOX7*-2 cells compared with A549-*SFTPC*-si-NC and H1299-*SFTPC*-si-NC cells, respectively (**Figures 3D, E**). Collectively, we demonstrated that overexpression of *SFTPC* significantly repressed migration, invasion abilities and EMT process of NSCLC cells via upregulating *SOX7*.

### 3.5 Overexpression of *SFTPC* inhibited EMT process of NSCLC cells through upregulation of *SOX7* and then inactivation of WNT/β-catenin pathway *in vitro*

**Figure 4:**
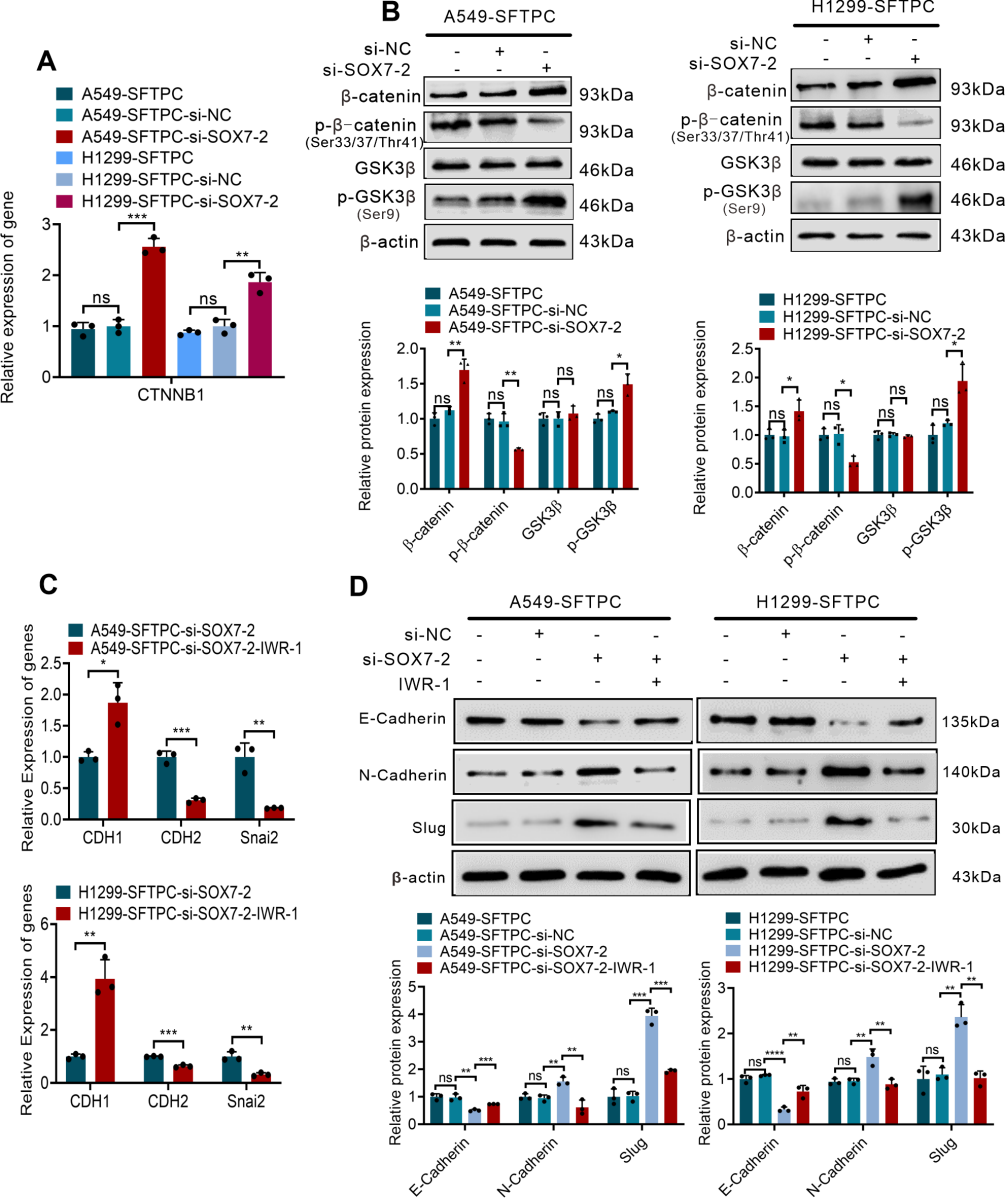
Overexpression of *SFTPC* inhibited EMT process through upregulation of *SOX7* and then inactivation of WNT/β-catenin pathway *in vitro*. **(A)** qRT-PCR assay was used to detect the mRNA level of *CTNNB1*(β-catenin). The histogram was applied to quantify the relative mRNA level. **(B)** Western blot assay was used to detect the protein levels of WNT/β-catenin pathway markers by using primary antibodies against GSK3β, p-GSK3β(Ser9), β-catenin, p-β-catenin (Ser33/Ser37/Thr41) in indicated cells, and the histograms were applied to quantify the relative protein levels. **(C)** qRT-PCR assay was conducted on the indicated cells treated with IWR-1 (0.5μM) or without IWR-1 (*n*=3), the relative mRNA levels of EMT markers (*CDH1*(E-cadherin), *CDH2*(N-cadherin), *Snai2*(Slug) were detected. The histograms were applied to quantify the relative mRNA levels. **(D)** Western blot assay was conducted on the indicated cells treated with IWR-1 (0.5μM) or without IWR-1 by using primary antibodies against E-cadherin, N-cadherin and Slug (*n*=3), and the histograms were applied to quantify the relative protein levels. β-actin was used as the internal control gene. All the data were presented as the Mean ± SD, un-paired *t*-test. *p<0.05, **p<0.01, ***p<0.005, ****p<0.001. ns, no significance. All experiments were conducted a minimum of three times independently.

Furthermore, to verify whether overexpression of *SFTPC* inhibited EMT process of NSCLC cells through upregulation of *SOX7* and then inactivation of WNT/β-catenin pathway, we firstly detected whether overexpression of *SFTPC* repressed WNT/β-catenin pathway via upregulating *SOX7*. Our results indicated the knockdown of *SOX7* in A549-*SFTPC* and H1299-*SFTPC* observably increased mRNA level of *CTNNB1*(β-catenin) by qRT-PCR (**Figure 4A**). Meanwhile, GSK3β was not changed, but p-GSK3β(Ser9) and β-catenin were notably increased while p-β-catenin (Ser33/Ser37/Thr41) was observably decreased in A549-*SFTPC*-si-*SOX7*-2 and H1299-*SFTPC*-si-*SOX7*-2 compared with A549-*SFTPC*-si-NC and H1299-*SFTPC*-si-NC cells by Western blot assay (**Figure 4B**). Taken together, overexpression of *SFTPC* could upregulate *SOX7*, then inhibit the expression of *CTNNB1*(β-catenin), reduce p-GSK3β(Ser9) and increase p-β-catenin (Ser33/Ser37/Thr41), which could lead to the decrease of β-catenin. Moreover, to clarify whether *SOX7* was the upstream factor of β-catenin, the inhibitor of WNT/β-catenin pathway was used in our experiment. IWR-1 had been proved to be a potent inhibitor of WNT/β-catenin pathway in a variety of cancer cells (37, 38).We found that the expression of SOX7 was not changed in A549-Control and H1299-Control cells after treatment with IWR-1 by qRT-PCR and Western blot assays (**Supplementary Figures S6A, B**). These outcomes indicated that overexpression of *SFTPC* inactivated WNT/β-catenin pathway though upregulating *SOX7*.

Then, based on the above results, IWR-1 was utilized in the cells where *SOX7* had been knocked down. The A549-*SFTPC*-si-*SOX7*-2 and H1299-*SFTPC*-si-*SOX7*-2 cells were treated by IWR-1 (named A549-*SFTPC*-si-*SOX7*-2-IWR-1 and H1299-*SFTPC*-si-*SOX7*-2-IWR-1). And *CDH1*(E-cadherin) was prominently increased, while *CDH2*(N-cadherin) and *Snai2*(Slug) were notably decreased in A549-*SFTPC*-si-*SOX7*-2-IWR-1 and H1299-*SFTPC*-si-*SOX7*-2-IWR-1 cells compared with A549-*SFTPC*-si-*SOX7*-2 and H1299-*SFTPC*-si-*SOX7*-2 cells by qRT-PCR and Western blot assays (**Figures 4C, D**). Taken together, we illustrated that overexpression of *SFTPC* obviously inhibited EMT process of NSCLC cells by upregulating *SOX7* and then repressing WNT/β-catenin pathway.

### 3.6 Overexpression of *SFTPC* inhibiting EMT process of NSCLC cells was associated with upregulation of *SOX7* and inactivation of WNT/β-catenin pathway *in vivo*

**Figure 5:**
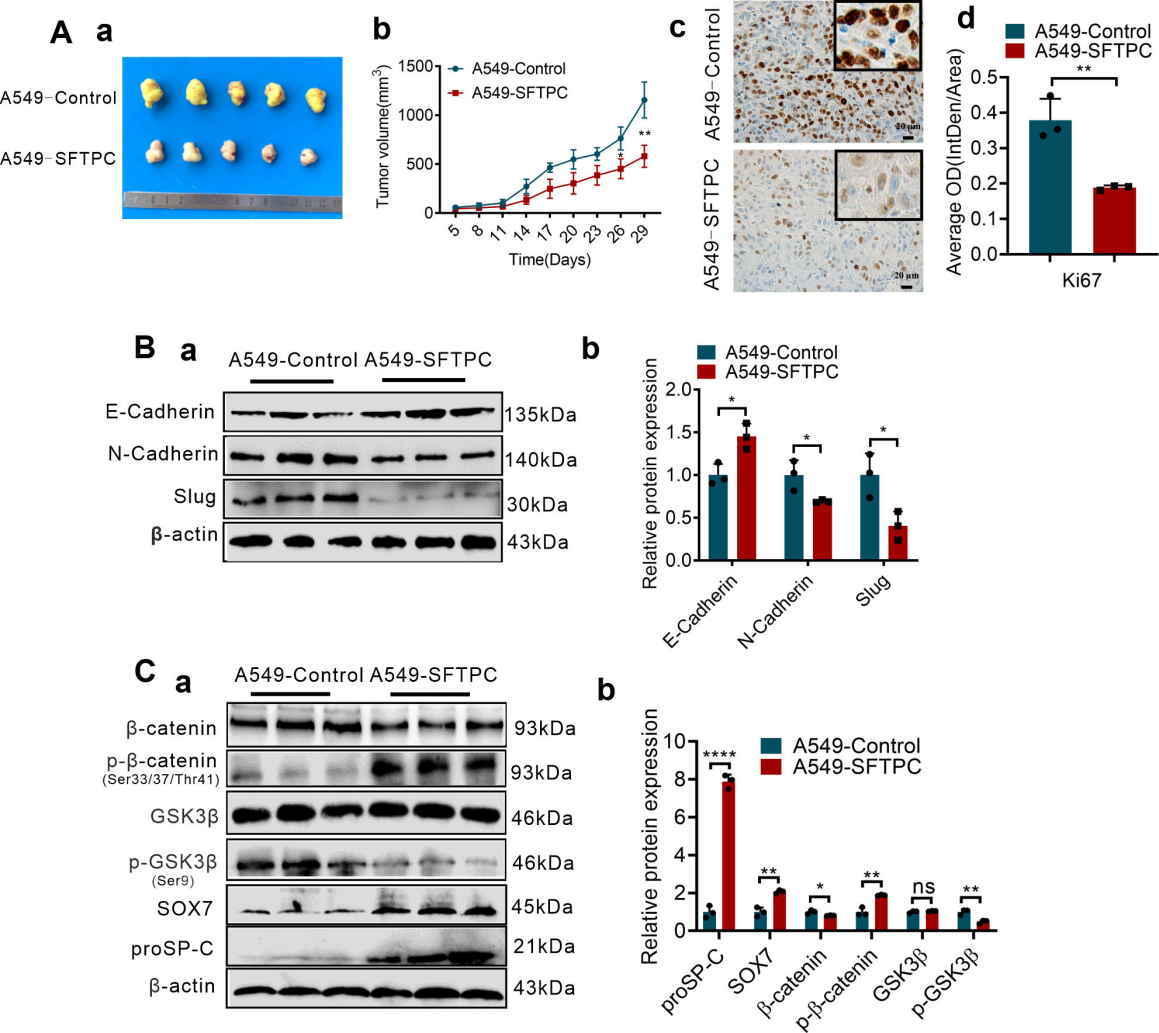
Overexpression of *SFTPC* inhibiting EMT process of NSCLC cells was associated with upregulation of *SOX7* and inactivation of WNT/β-catenin pathway *in vivo*. **(A)** Establishment of stable *SFTPC*-overexpression A549 and control tumor xenograft models. BALB/c nude mice (4-week-old male, *n*=5/group) were subcutaneously injected with A549-*SFTPC* cells or A549-Control cells, respectively. The mice were sacrificed after 29 days, the retrieved tumor samples were showed in (a). The statistical graph of changes in tumor volume were showed in (b). IHC staining was conducted on paraffin sections of retrieved tumor samples by using primary antibody against the proliferation marker Ki-67 (*n*=3) (c), staining without primary antibody was used as negative control. Image J 180 was used to measure the average optical density. The histogram was applied to quantify the results of IHC staining (d). Scale bars, 20μm. **(B)** Western blot assay was conducted on the protein of retrieved tumor samples (*n*=3) by using the primary antibodies against E-cadherin, N-cadherin and Slug (a), and the histogram was applied to quantify the relative protein levels (b). **(C)** Western blot assay was conducted on the protein of the retrieved tumor samples (*n*=3) by using the primary antibodies against SOX7, proSP-C, GSK3β, p-GSK3β(Ser9), β-catenin and p-β-catenin (Ser33/Ser37/Thr41) (a), the histogram was applied to quantify the relative protein levels (b). β-actin was used as the internal control gene. All data were presented as the Mean ± SD, un-paired *t*-test. *p<0.05, **p<0.01, ****p<0.001. All experiments were conducted a minimum of three times independently.

To further confirm whether overexpression of *SFTPC* inhibiting EMT process was related to upregulation of *SOX7* and inactivation of WNT/β-catenin pathway *in vivo*, the stable *SFTPC*-overexpression A549 cells tumor xenograft model and its control model were established via subcutaneous injection of A549-*SFTPC* and A549-Control cells according to our previous study (25). Experimentally, five days after cells were injected subcutaneously, the tumor formed, then, all tumors size was measured every three days. Compared to A549-Control tumors, the tumors volume was observably inhibited and the cell proliferation marker Ki-67 was notably decreased in A549-*SFTPC* tumors (**Figure 5A**). Importantly, compared to control tumors, E-cadherin was notably increased while N-cadherin and Slug were dramatically decreased in A549-*SFTPC* tumors by Western blot assay (**Figure 5B**). Moreover, the level of GSK3β was not changed, and the levels of proSP-C, SOX7 and p-β-catenin (Ser33/Ser37/Thr41) were markedly increased, while the levels of p-GSK3β(Ser9) and β-catenin were visibly decreased in A549-*SFTPC* tumors compared with A549-Control tumors by Western blot analysis (**Figure 5C**). All the outcomes *in vivo* were consistent with their results *in vitro*. It illustrated that overexpression of *SFTPC* inhibiting EMT process of NSCLC cells was relevant to upregulating *SOX7* and repressing WNT/β-catenin pathway in tumor xenograft models.

### 3.7 Low expression of *SFTPC* was correlated with EMT process and low expression of *SOX7* in collected clinical LUAD tissues

**Figure 6:**
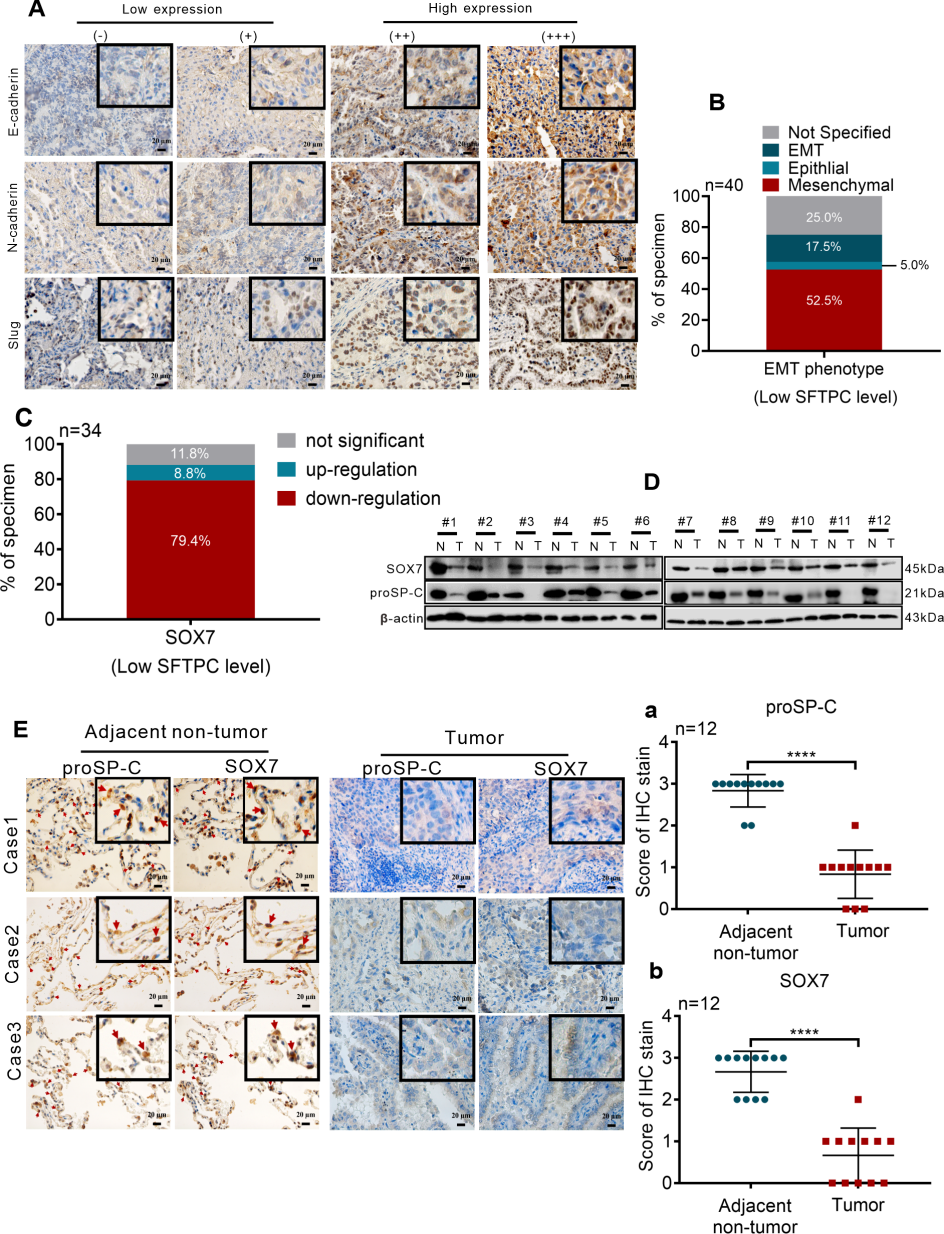
Low expression of *SFTPC* was correlated with the EMT process and low expression of *SOX7* in clinical LUAD tissues **(A)** IHC staining was conducted on paraffin sections of collected clinical LUAD tumor tissues with low mRNA level of *SFTPC* (*n*=40) by using primary antibodies against E-Cadherin, N-Cadherin and Slug. Staining without primary antibody was used as negative control. The IHC staining yielded grades of E-Cadherin, N-Cadherin and Slug as follows, (-) and (+) represented low expression of EMT markers. (++) and (+++) represented high expression of EMT markers. Scale bar, 20μm. **(B)** The relationship between low expression of *SFTPC* and EMT phenotype status in LUAD tissues. **(C)** The mRNA levels of *SOX7* in the collected clinical LUAD tumor tissues with low mRNA levels of *SFTPC* (*n*=34) were detected by qRT-PCR assay. **(D)** Western blot assay was conducted on the protein of paired clinical LUAD tumor tissues with both low mRNA levels of *SFTPC* and *SOX7* (*n*=12) by using the primary antibodies against SOX7 and proSP-C. **(E)** IHC staining was conducted on the paraffin sections of the collected paired clinical LUAD tumor tissues with both low mRNA levels of *SFTPC* and *SOX7* (*n*=12) by using primary antibodies against proSP-C and SOX7. Staining without primary antibody was used as negative control. And the site of SOX7 in alveolar (indicated by red arrow, adjacent non-tumor groups, right) was detected by IHC staining (*n*=12). The histograms were applied to quantify the experimental results of IHC staining (a, b). *n*, numbers of clinical tissues. Scale bar, 20μm. All data were presented as the Mean ± SD, un-paired *t*-test. ****p<0.001.

To further clarity the correlation of *SFTPC* and EMT phenotype in clinical NSCLC tissues, the protein levels of EMT markers in 40 cases of clinical LUAD tissues with low expression of *SFTPC* were detected by IHC staining. According to our previous study (25), the EMT phenotype of tumors was distinguished though the protein levels of EMT markers. Therefore, the EMT phenotype were classified based on the defined criteria in present study (**Figure 6A**). In 40 cases of clinical LUAD tissues, the ratio of mesenchymal (*n*=21, 52.5%) and EMT (*n*=7, 17.5%) phenotypes were significantly higher than the ratio of epithelial (*n*=2, 5.0%) phenotype (**Figure 6B**, **Supplementary Table S5**). It demonstrated that low expression of *SFTPC* was correlation with EMT process of clinical NSCLC samples.

Then, to further investigate the relationship between *SFTPC* and *SOX7* in NSCLC samples, the expression changes of *SOX7* in 34 cases of clinical LUAD tissues with low expression of *SFTPC* were detected by qRT-PCR assay. Compared to paired adjacent non-tumor tissues, the mRNA levels of *SOX7* were notably decreased in 27 out of 34 LUAD tissues (**Figure 6C**, **Supplementary Table S6**). Then, 12 pairs of LUAD were further selected from the 27 pairs of LUAD with both low mRNA levels of *SFTPC* and *SOX7* randomly. The protein levels of proSP-C and SOX7 were both downregulated in the 12 pairs of LUAD by IHC staining and Western blot assays (**Figures 6D, E**). The score criteria and outcomes for proSP-C and SOX7 of IHC staining were showed in **Supplementary Table S2**. Moreover, we discovered that SOX7 and proSP-C protein were colocalized in AT2 cells in adjacent non-tumor tissues by IHC staining (**Figure 6E**). The outcomes above indicated that there was a positive relationship between *SFTPC* and *SOX7* in LUAD tissues. In total, we proved that low expression of *SFTPC* was correlated with the EMT process and low expression of *SOX7* in clinical LUAD tissues.

## 4 Discussion

The functions and mechanisms of AT2 cells marker gene *SFTPC* in NSCLC development are still poor understood until now. In our present study, we demonstrated that overexpression of *SFTPC* repressed EMT process of NSCLC cells via upregulating *SOX7* and then inactivating WNT/β-catenin pathway for the first time.

Firstly, we discovered that the expression of *SFTPC* was extremely decreased in 515 LUAD cases compared with 59 adjacent non-tumors in TCGA database. Subsequently, we further clarified that the expression of *SFTPC* was observably decreased in 40 out of 46 collected LUAD samples compared with matched adjacent non-tumors. Analogously, some researchers also discovered that the expression of *SFTPC* was notably downregulated in NSCLC samples compared with the adjacent non-tumors (24, 27, 28). Consistent with the results of others, our current research further proved that the expression of *SFTPC* was remarkably decreased in NSCLCs compared with the adjacent non-tumors.

Subsequently, we further found that low expression of *SFTPC* in NSCLC tissues was related to lymph node metastasis of LUAD, and was also related to low OS rate of LUAD patients in TCGA database. Unfortunately, in the 45 cases of collected LUAD samples, the expression level of *SFTPC* had no significant difference between the LUAD samples with lymph node metastasis and without lymph node metastasis. It might be limited by the number of LUAD samples, and it will be further analyzed in more LUAD samples in the future. It is worth mentioning that Li Wang et al. (39) uncovered that the expression of *SFTPC* was progressively downregulated during the malignant progression of LUAD, and it was completely absent in LUAD tissues with brain metastases. Similarly, the survival analysis conducted by multiple researchers across multiple databases suggested that low expression of *SFTPC* was observably correlated with the decreased OS rate and progression-free survival (FP) rate of NSCLC patients (24, 28, 40, 41). Taken together, our results and others all indicated that low expression of *SFTPC* was associated with poor prognosis of NSCLC.

Next, we found that overexpression of *SFTPC* significantly repressed the migration and invasion abilities of NSCLC cells. Importantly, we further proved that overexpression of *SFTPC* dramatically repressed EMT process of NSCLC cells. Previously, Bin Li et al. (29) and Baile Zuo et al. (24) only found that overexpression of *SFTPC* inhibited the proliferation ability of NSCLC cells. In comparison, our study further enriched the functions of *SFTPC* in the malignant progression of NSCLC. Especially, we found low expression of *SFTPC* was correlation with EMT process in clinical NSCLC tissues. Of note, EMT process of NSCLC cells promoted metastasis of tumors (13, 23), and low expression of E-cadherin was markedly relevant to poor prognosis of LUAD (23). Therefore, our results also implied that *SFTPC* might be a potential prognostic marker gene for NSCLC. Then, we discovered that overexpression of *SFTPC* could prominently upregulate the expression of SOX7 in NSCLC cells through RNA-seq, qRT-PCR and Western blot assays. SOX7 protein belongs to SOX family, which are transcription factors (42). It was demonstrated that *SOX7* was a tumor suppressor in lung cancer (31, 33). Previous studies discovered that the downregulation of *SOX7* in lung tumor tissues could be caused by hypermethylation in its promoter (43). What’s more, the expression of *SOX7* could also be downregulated or upregulated by miRNA, lncRNA or GATA4 (36). Here, our study found that overexpression of *SFTPC* upregulated the expression of *SOX7.* Our results further enriched the upstream regulatory network of *SOX7*. Of course, we didn’t clarify the underlying molecular mechanism about overexpression of *SFTPC* upregulating *SOX7*, and it would be an important part in our subsequent study.

Strikingly, several studies illustrated that low expression of *SOX7* in LUAD tissues was related to lymph node metastasis and low OS rate of LUAD patients (30, 31). And Lichun Han et al. (33) proved overexpression of *SOX7* inhibited the malignant progression of NSCLC. Here, we found that knockdown of *SOX7* enhanced the migratory and invasive abilities of A549-*SFTPC* and H1299-*SFTPC* cells. Meanwhile, a typical spindle-like shape change had been observed in A549-*SFTPC*-si-*SOX7*-2 cells. And knockdown of *SOX7* significantly downregulated E-cadherin while notably upregulated N-cadherin and Snai2 in A549-*SFTPC* and H1299-*SFTPC* cells. Collectively, our results above confirmed that overexpression of *SFTPC* repressed EMT process of NSCLC cells via upregulating *SOX7*, thereby suppressed their migration and invasion abilities.

Furthermore, our RNA-seq result suggested that overexpression of *SFTPC* was negatively associated with WNT/β-catenin pathway, then we demonstrated that overexpression of *SFTPC* prominently inactivated WNT/β-catenin pathway by qRT-PCR and Western blot assays. It is worth mentioning that Lizheng Guo et al. (35) uncovered that SOX7 protein interacted with β-catenin directly, which mediated the degradation of active β-catenin in the APC-independent mechanism. Here, we found knockdown of *SOX7* in A549-*SFTPC* and H1299-*SFTPC* cells observably increased the mRNA level of *CTNNB1*(β-catenin), the protein levels of p-GSK3β(Ser9) and β-catenin, but decreased the level of p-β-catenin (Ser33/Ser37/Thr41) compared with control cells. Taken together, we demonstrated that overexpression of *SFTPC* inactivated WNT/β-catenin pathway via upregulation of *SOX7*. However, the mechanism about overexpression of *SFTPC* upregulating *SOX7* inhibited the expression of *CTNNB1*(β-catenin) didn’t been further explored in our present study. It will be clarified thorough further research in the future. Interestingly, Yumeng Zhang et al. (44) found that overexpression of *SOX7* could directly activate *SLIT2* in human breast cancer MDA-MB-231 cells. Meanwhile, we found that *SLIT2* was upregulated in A549-*SFTPC* group **Supplementary Figure S3D-b**). Importantly, Dinesh K. Ahirwar et al. (45) found that overexpression of *SLIT2* inhibiting small cell lung cancer growth was associated with the inactivation of the GSK3β/β-catenin signaling pathway in tumor cells and tumor-associated macrophages. In our present study, we didn’t clarify the mechanism about overexpression of *SFTPC* upregulating *SOX7* inhibited the protein level of p-GSK3β(Ser9) in NSCLC cells. Therefore, based on our and others’ research, we can hypothesize that overexpression of *SFTPC* upregulating *SOX7* might directly activate *SLIT2*, and then inhibit the protein level of p-GSK3β(Ser9) in NSCLC cells. And this hypothetical will be an essential part in our subsequent research.

WNT/β-catenin pathway, which promoted the formation of airway and alveoli, played critical role in pulmonary development (34). The hyperactivation of this pathway was thought to be a vital factor of EMT process (13). We discovered that E-cadherin was notably increased while N-cadherin and Snai2 were markedly decreased in A549-*SFTPC*-si-*SOX7*-2 and H1299-*SFTPC*-si-*SOX7*-2 cells after treatment with IWR-1, the potent inhibitor of WNT/β-catenin pathway. The outcomes above confirmed that overexpression of *SFTPC* repressed EMT process of NSCLC cells via upregulation of *SOX7* and then inactivation of WNT/β-catenin pathway.

Particularly, overactivation of WNT/β-catenin pathway was found in AT2 cells when alveoli were severely injured (5), and overactivation of this pathway was tightly linked with malignant progression of lung cancer (34). Importantly, abnormal AT2 cells were one of the origin cells of NSCLC (17). However, it was difficult to explore the molecular mechanisms about how NSCLC cells could derive from malignant transformation of the AT2 cells directly, since AT2 cells would rapidly differentiate into AT1 cells in 2D culture *in vitro (* 46). Here, we found overexpression of *SFTPC* repressed EMT process of NSCLC cells via upregulating *SOX7* and then inactivating WNT/β-catenin pathway. It might provide a new idea for further investigating the mechanisms about the malignant transformation of AT2 cells into NSCLC cells in the future. Importantly, Hsin-Jung Li et al. (47) discovered that the inactivation of WNT/β-catenin pathway inhibited EMT process and metastasis of NSCLC recently. Moreover, some inhibitors of WNT/β-catenin pathway were investigated in a series of NSCLC clinical trials (48). Therefore, our results suggested that *SFTPC* might also be a novel target for clinical NSCLC treatment.

In addition, several studies found that mutations or deletions in *SFTPC* caused IPF (8, 12, 49). Strikingly, Liudi Yao et al. (50) discovered that abnormal gene in AT2 cells induced EMT progress of AT2 cells, then promoted IPF development. The epidemiological researches reported IPF was also an independent risk factor for lung cancer (19). Importantly, major lung tumors (70%~82%) of the patients with IPF were located near fibrotic lesions by pathological studies (51, 52). However, the mechanisms about lung tumor development in patients with IPF are still poorly understood. Interestingly, Takayuki Honda et al. (53) found *SFTPC* mutation in patients with both IPF and NSCLC (IPF-NSCLC) by Whole-exome analysis, and revealed that *SFTPC* mutation could notably decrease the expression of *SFTPC*. Here, we proved the expression of *SFTPC* was extremely decreased in clinical NSCLC samples, and we clarified that overexpression of *SFTPC* repressed EMT process of NSCLC cells via upregulating *SOX7* and then inactivating WNT/β-catenin pathway. Collectively, our study suggested that the abnormal expression of *SFTPC* might induce EMT process of AT2 cells, then promote the development of IPF and NSCLC.

**Figure 7:**
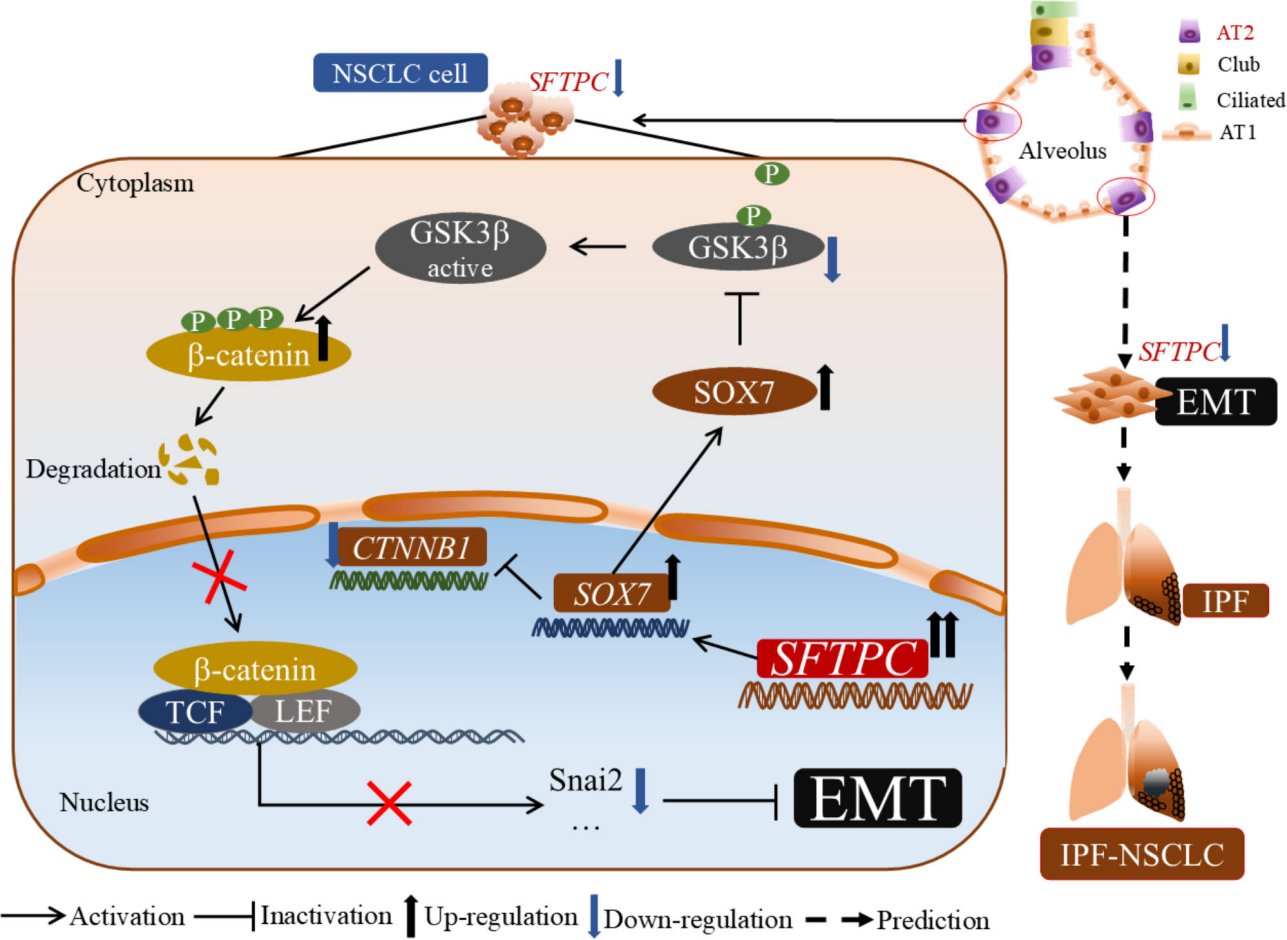
The molecular model of overexpression of *SFTPC* inhibiting the EMT process of NSCLC cells. Mechanistically, overexpression of *SFTPC* upregulated the expression of *SOX7*, then inhibited the mRNA level of *CTNNB1*(β-catenin) and decreased the protein level of p-GSK3β(Ser9), whereas increased the protein level of p-β-catenin (Ser33/Ser37/Thr41), and further decreased the protein level of β-catenin, and inhibited the EMT process of NSCLC cells eventually. In addition, we further speculated that the abnormal expression of AT2 cells marker gene *SFTPC* might induce EMT process in AT2 cells, then promote the development of IPF-NSCLC. (This figure was drawn by power point with Science Slides suite).

In conclusion, our results revealed a new molecular mechanism of the AT2 cells marker gene *SFTPC* in development of NSCLC. Moreover, our research might also provide a novel clue for exploring the molecular mechanism about NSCLC development in patients with IPF in the future (**Figure 7**).

## Data Availability

The RNA-seq datasets presented in this study can be found in online repositories. The names of the repository/repositories and accession number(s) can be found below: http://www.ncbi.nlm.nih.gov/bioproject/1157334, BioProject ID: PRJNA1157334. Further inquiries can be directed to the corresponding authors.
