# Supplementary material for: Alveolar type 2 cells marker gene SFTPC inhibits epithelial-to-mesenchymal transition by upregulating SOX7 and suppressing WNT/β-catenin pathway in non-small cell lung cancer
Source: Front Oncol. 2024 Sep 13;14:1448379. doi: 10.3389/fonc.2024.1448379 (PMC11427448; doi:10.3389/fonc.2024.1448379)
Supplement: Supplementary file 1 [file DataSheet1.pdf]

Supplementary Material

1 Supplementary Materials and Methods

1.1 NSCLC patient samples

All the 46 pairs of clinical LUAD tissues were obtained from West China Hospital, Sichuan University and Sichuan Provincial People's Hospital (Chengdu, China). Clinical information of the 46 LUAD cases were presented in Supplemental Table S1.

1.2 Bioinformatics

Two different websites about The Cancer Genomics Altas (TCGA) database, UALCAN (http://ualcan.path.uab.edu/index.html) and GEPIA (http://gepia2.cancer-pku.cn/#index), were used in this part. In total, 515 LUAD cases and 59 normal lung tissues got from UALCAN were used to measure the expression of *SFTPC* and analyze the relationship between *SFTPC* and lymph node metastasis of LUAD. Meanwhile, 240 LUAD cases were performed by GEPIA to preliminarily analyze the correlation between the expression of *SFTPC* and overall survival (OS) rate.

1.3 Cell lines and cultures

The cells were used after authentication by morphologic inspection as described in previous study of our group(1) and were detected for *Mycoplasma* using *Mycoplasma* Delector Kit (qRT-PCR-TaqMan) (CWBIO, Cat. CW3026S). All the human NSCLC cells were cultured in RPMI-1640 medium (Invitrogen, Carlsbad, CA, USA) supplemented with 10% fetal bovine serum (Invitrogen/Gibco), 100 units of penicillin and 100 µg of streptomycin at 37 ^{o}C in 5% CO_2. Recombinant lentiviruses expressing *SFTPC* (Gene ID: 6440) and the control lentiviruses were constructed and packaged by HanBio (Shanghai, China). The stable *SFTPC*-overexpressing A549 and H1299 cell lines and their control cell lines were established by lentivirus infection and puromycin (1µg/mL) (Merck Millipore, Burlington, MA, USA) selection as previously described(1). Lipofectamine 3000 (Invitrogen, Carlsbad, CA) was used to transfect cells with si-*SOX7* which was synthesized by HanBio (Shanghai, China). 0.5µM IWR-1(Selleckchem, #S7086) was used to inhibit WNT/β-catenin pathway. Total RNA and proteins were extracted from 90% confluent cells in culture dishes. The sequences of the si-*SOX7*-1, si-*SOX7*-2 and si-*SOX7*-3 were shown in Supplemental Table S7.

1.4 RNA-sequence (RNA-seq)

RNA was extracted, sequenced, and analyzed by custom service provided by Genedenovo Biotechnology Co., Ltd (Guangzhou, China) using the Illumina Novaseq6000 platform. The transcriptome data used in the present study were raw count and FPKM (Fragments per kilobase of transcript sequence per millions base pairs sequenced). Differential expression analysis between two groups (A549-*SFTPC* vs. A549-Control) was performed by using the DESeq2 R package. Significant differentially expressed genes (DEGs) between groups (n = 3/group) were determined by false discovery rate (FDR)<0.05 and log2|FC|>1. Gene Ontology (GO) analysis, KEGG analysis and Reactome analysis were applied to determine the functions of those DEGs by GO (http://www.geneontology.org/), KEGG (KEGG: Kyoto Encyclopedia of Genes and Genomes), and

Reactome (https://reactome.org/). Gene Set Enrichment Analysis (GSEA) analysis was used to provide a comprehensive interpretation of the regulatory role of a functional unit (GO term, KEGG pathway or Reactome term). In addition, for the screening of the downstream genes of *SFTPC*, the datasets using in our study could obtain from two different websites for the TCGA database, GEPIA "http://gepia2.cancer-pku.cn" and UALCAN "http://ualcan.path.uab.edu/analysis.html" . Crossover analysis of the two datasets and the DEGs got from RNA-seq was performed on the Veen Map online rendering website "http://bioinfogp.cnb.csic.es/tools/venny/index.html".

1.5 Quantitative real-time PCR (qRT-PCR)

In this part, the total mRNA of all cells and freshly-prepared clinical NSCLC tissues were obtained by using the TRIZOL Reagent (Invitrogen, China) and then reverse-transcribed into cDNA products by using Hifair® III 1st Strand cDNA Synthesis SuperMix for qRT-PCR (gDNA digester plus) (YEASEN, Shanghai, China). 2× SYBR Green qPCR Master Mix (YEASEN, Shanghai, China) was utilized in qRT-PCR assay. The experiment was conducted on the Light Cycler 96 (Roche, Shanghai, China). Sequences of the qRT-PCR primers were shown in Supplemental Table S8.

1.6 Western blot

Total protein was got from lysate of indicated cells, clinical tissues or retrieved subcutaneous tumor tissues. The proteins were probed with primary antibodies against β-actin (1:1000 dilution, Abcam, Cat. #ab8226), proSP-C (1:1000 dilution, Abcam, Cat. #ab90135), SOX7 (1:1000 dilution, Proteintech, Cat. #23925-1-AP), β-catenin (1:1000 dilution, CST, Cat. #9562), p-β-catenin (Ser33/Ser37/Thr41) (1:1000 dilution, CST, Cat. #9561p), GSK3β (1:1000 dilution, CST, Cat. #12456s), p-GSK3β(Ser9) (1:1000 dilution, ZEN-BIOSCIENCE, Cat. #310010), E-cadherin (1:1000 dilution, Affinity, Cat. #AF0131), N-cadherin (1:1000 dilution, Affinity, Cat. #AF4039), Slug (1:1000 dilution, Affinity, Cat. #DF6202). All experiments were conducted a minimum of three times independently, and the results of three independent experiments were evaluated by Image J 180 software and statistical analysis was performed by GraphPad Prism 9.5 software.

1.7 Immunohistochemical (IHC) staining

For IHC staining, the clinical tissues and retrieved subcutaneous tumor tissues were fixed in fresh 4% paraformaldehyde overnight, paraffin embedded and sectioned. The sections were subjected to deparaffinization, followed by antigen retrieval and immunostaining with different antibodies against proSP-C (1:100 dilution, Abcam, Cat. #ab90135), Ki-67(1:200 dilution, Abcam, Cat. #ab15580), SOX7 (1:100 dilution, Proteintech, Cat. #23925-1-AP), E-cadherin (1:100 dilution, Affinity, Cat. #AF0131), N-cadherin (1:100 dilution, Affinity, Cat. #AF4039), Slug (1:100 dilution, Affinity, Cat. #DF6202). Stains without primary antibody were used as negative control. Examined and recorded under a microscope (magnification, 400×).

For the IHC score which included adjacent non-tumor tissues, we just used the staining intensity score for the IHC staining(2). But for the EMT phenotype assessment, the IHC score (Q-score) and EMT phenotype criteria for NSCLC tissues were defined based on the previous study(1). For the Q-score (staining intensity score × proportion of positive cells score), the staining intensity was graded as 0, negative staining; 1, weak staining; 2, moderate staining; and 3, strong staining. The proportion of positively stained cells per specimen was determined as follows: 0 for no positively stained cells; 1 for <10%; 2 for 10–50%; and 3 for >50% of the examined cells. Q-score ≥4 was considered high

expression, and ≤3 was considered low expression. A final total score was graded as negative (−), weak (+), moderate (++), or strong (+++). For the tissues with high expression of E-cadherin and low expression of all mesenchymal phenotype markers, the phenotype of them was defined as epithelial phenotype. For the tissues with high expression of one of the mesenchymal phenotype markers together with low expression of E-cadherin, the phenotype of them was defined as mesenchymal phenotype. For the tissues with high expression of both E-cadherin and mesenchymal phenotype markers, the phenotype of them was defined as EMT phenotype. And the remaining were not specified.

1.8 Wound-healing assay

When cells were grown to confluence, draw a straight linear scratch on the cell monolayers by using a 200μL pipette tip. To remove cells from the cell cycle prior to wounding, cells were maintained in serum-free medium. To visualize migrated cells and wound healing, images were taken at 0, 24 hours under a Zeiss Imager Z2 microscope (Carl Zeiss, Oberkochen, Germany) (50×). Three areas were selected from each group quantified randomly.

1.9 Millcell and Transwell assays

The Transwell chambers (8mm pore size, Millipore, Switzerland) were coated with Matrigel (BD Bio-sciences, USA) diluted with RPMI-1640 (1:5) on the upper chamber in advance. Each Millicell chamber contained about 3×10^4 cells and each Transwell chamber contained about 5×10^4 cells. The chambers suspended on 24 well plate. Media containing 10% FBS was added to the lower chamber. Cells cultured at 37 °C incubator for 24h, then fixed in 4% paraformaldehyde, the non-migrating cells or the non-invading cells on the upper of the chambers were removed with cotton wool and PBS, migratory cells or invasive cells located on the lower surface of the chamber were stained by Crystal Violet Stain (Sigma Diagnostics; St Louis, Missouri, USA). Photographs of migrated or invaded cells were taken with the Zeiss Imager Z2 microscope (Carl Zeiss, Oberkochen, Germany) (200×). These assays were conducted at least three times independently.

1.10 Tumor xenograft model

All the ten 4-weeks-old BALB/c male nude mice were purchased from HFK Bioscience (Beijing, China), and were divided into two groups to create the xenograft tumor model randomly (the two groups: A549-*SFTPC* and A549-Control, five mice/group). The stable *SFTPC*-overexpression A549 cells and control cells were digested to obtain single cell suspension. Cells were subcutaneously implanted into the shoulder of mice. Each nude mouse was subcutaneously injected with 100μL medium containing about 5×10^6 cells. The tumor size was measured every three days after 5 days injection. All the mice were euthanized after 29 days. The volume of tumor (mm^3) was calculated by: Volume = length × width^2 × π/6. The half of all the transplanted tumors were placed in 4% paraformaldehyde and then embedded with paraffin for IHC staining, the half of tumors were used to Western blot.

2 Supplementary Figures and Tables

Figure S1. The establishment of the stable *SFTPC*-overexpressing NSCLC cell lines.

Figure S2. The number and hierarchial clustering heatmap of DEGs from RNA-seq

Figure S3. The bioinformatics analysis of DEGs.

Figure S4. The GSEA analysis of RNA-seq data.

Figure S5. The si-RNA-mediated knockdown of *SOX7* in stable *SFTPC*-overexpression NSCLC cells.

Figure S6. The detection of expression level of SOX7 in the cells after treatment with IWR-1.

Supplementary Table S1. The clinicopathological features and relative expression of *SFTPC* in 46 LUAD tissues.

Supplementary Table S2. The IHC staining score criteria and outcomes of proSP-C and SOX7.

Supplementary Table S3. Expression of *SFTPC* in LUAD based on nodal metastasis status in TCGA database

Supplementary Table S4. The correlation of the expression of *SFTPC* and age, gender, T stage, lymph node metastasis in 45 LUAD tissues.

Supplementary Table S5. The relationship between low expression of *SFTPC* and EMT phenotype status in LUAD patients.

Supplementary Table S6. The expression levels of *SOX7* in LUAD tissues with low expression of *SFTPC*.

Supplementary Table S7. The sequences of si-RNA.

Supplementary Table S8. The sequences of genes for qRT-PCR

2.1 Supplementary Figures

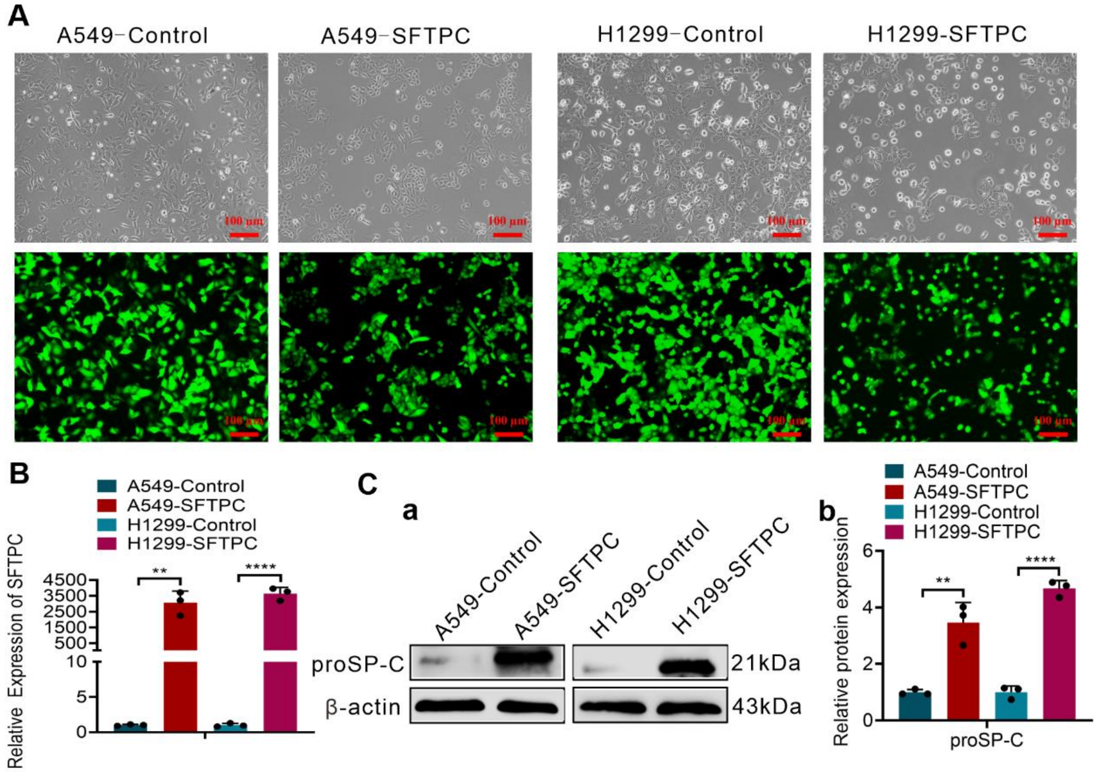

Supplementary figure 1. The establishing of stable *SFTPC*-overexpression NSCLC cell lines.

(A). The fluorogram of A549 and H1299 cells after lentivirus infection treatment. **Green fluorescent** cells represented the successful establishment of the stable *SFTPC*-overexpression NSCLC cells and their control cells. Scale bar, 100μm. **(B).** The mRNA levels of *SFTPC* in stable *SFTPC*-overexpression NSCLC cells and control cells were detected by qRT-PCR assay. **(C).** The protein levels of proSP-C in stable *SFTPC*-overexpression NSCLC cells and control cells were detected by Western blot assay. The data were presented as the Mean ± SD, un-paired t-test. **p<0.01, ****p<0.001. β-actin was used as the internal control gene.

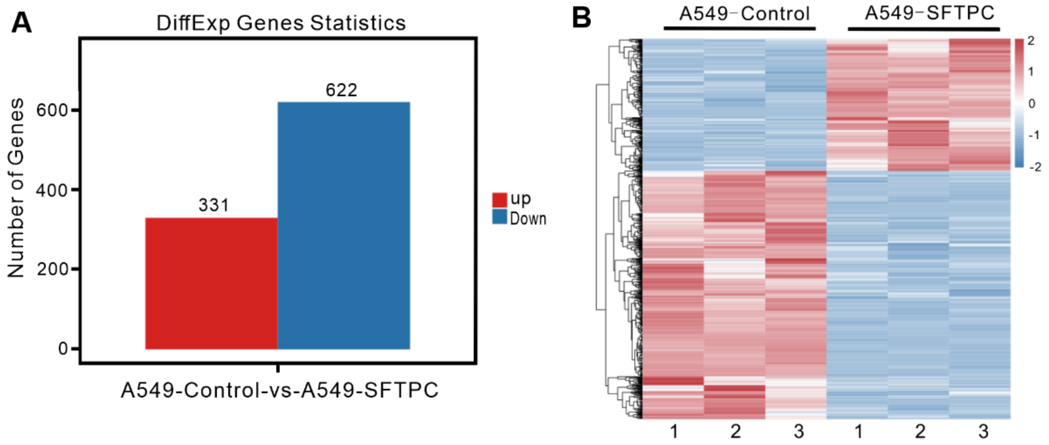

Supplementary figure 2. **The number and hierarchial clustering heatmap of DEGs from RNA-seq.**

(A). The number of differentially expressed genes (DEGs) between A549-*SFTPC* cells and A549-Control cells. Significant DEGs between two groups (n = 3/group) were determined by FDR<0.05 and log2|FC|>1. **(B)**. The hierarchial clustering heatmap of the DEGs. FDR<0.05 and log2|FC|>1.

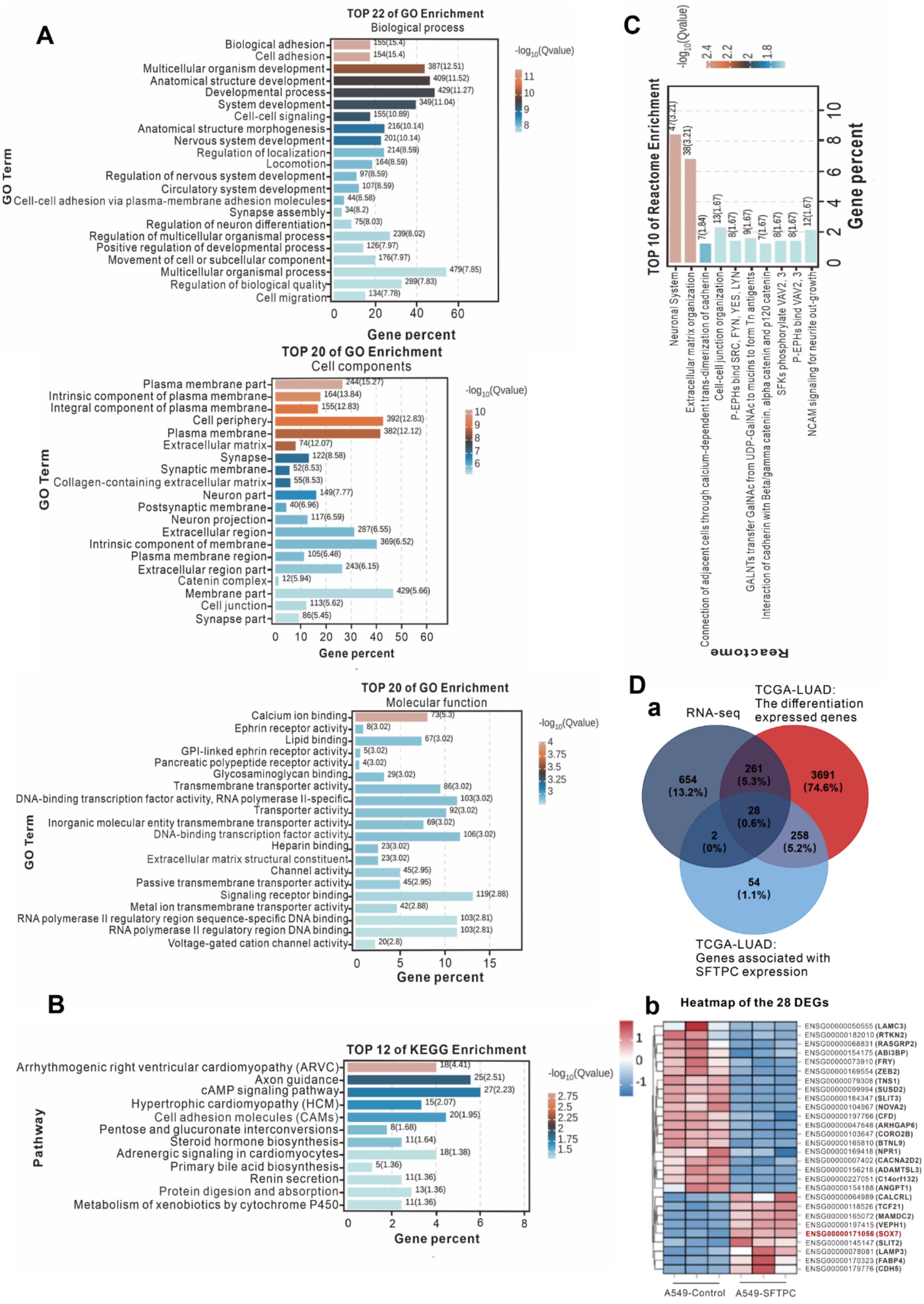

Supplementary figure 3. The bioinformatics analysis of DEGs.

(A). The GO analysis of DEGs, the ontologies of GO were biological process**,** cell components and molecular function. **(B).** the KEGG analysis of DEGs. **(C)**. Reactome analysis of DEGs. **(D)**. Venn diagram of the number of potential target genes of *SFTPC* by three datasets: “the differentially expressed genes in LUAD”, “genes associated with *SFTPC* in LUAD” and the DEGs got from RNA-seq (**a**). And the heatmap of the 28 overlapping DEGs from this Venn diagram (**b**).

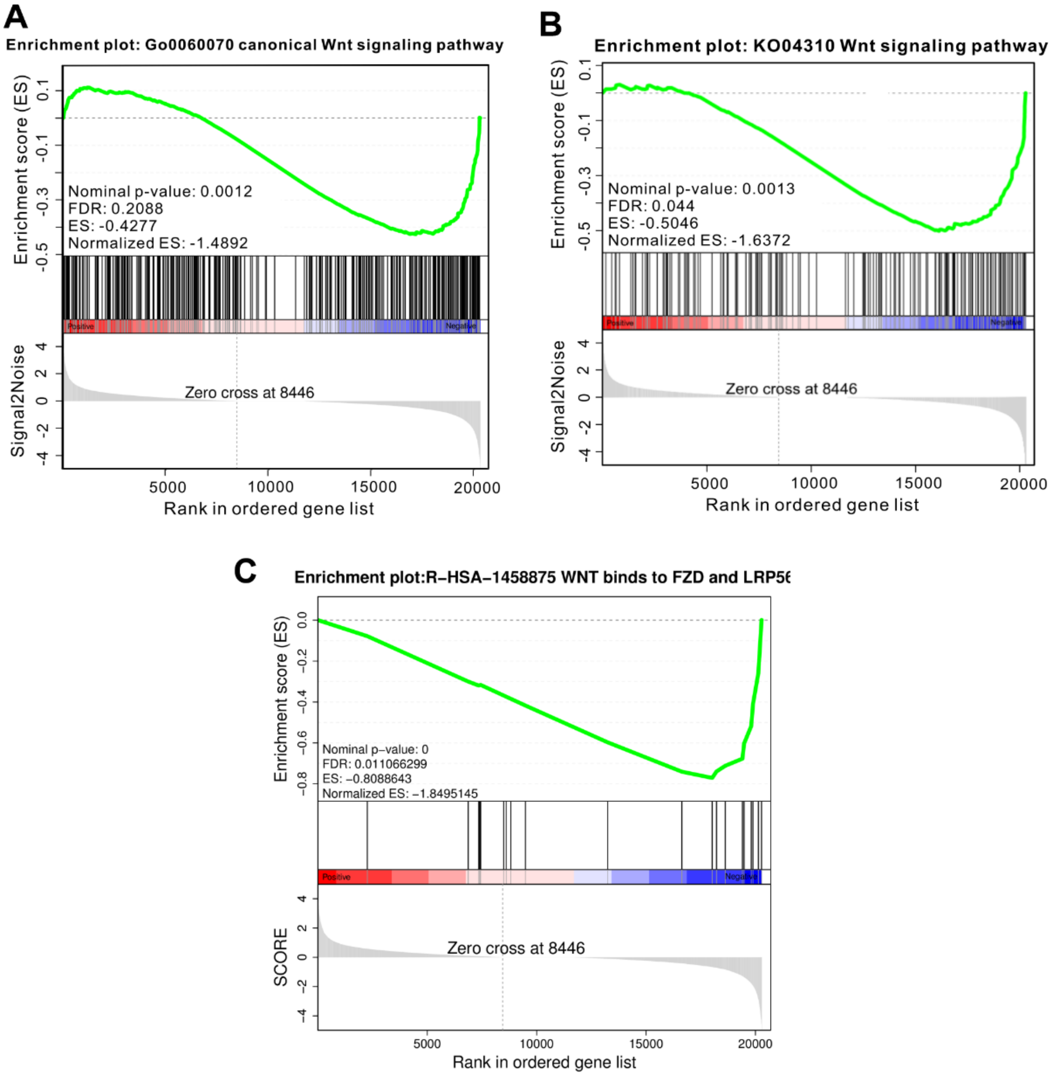

Supplementary figure 4. **The GSEA analysis of RNA-seq data.**

The GSEA-GO **(A)**, GSEA-KEGG **(B)** and GSEA-Reactome **(C)** analysis of RNA-seq data.

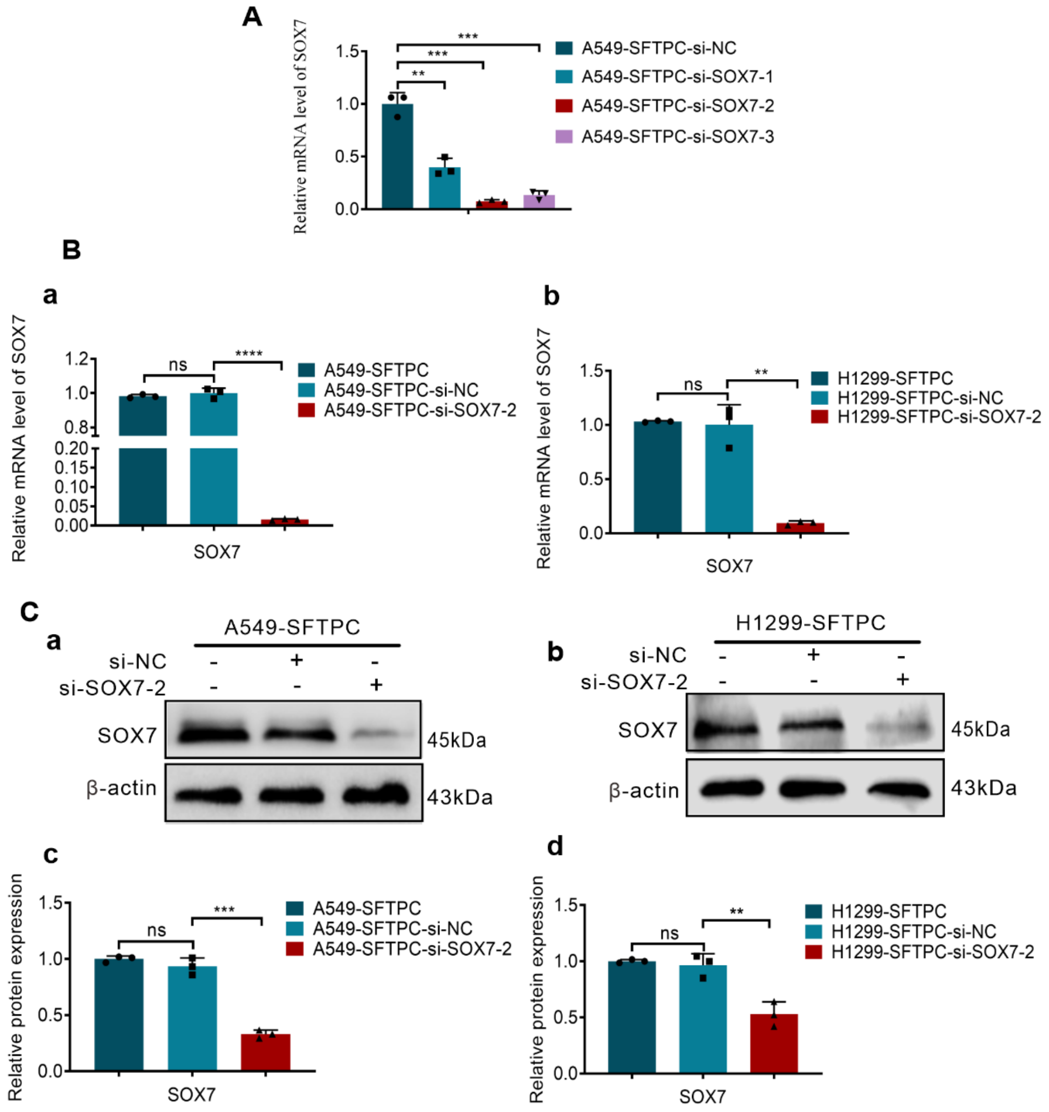

Supplementary figure 5. The si-RNA-mediated knockdown of *SOX7* in stable *SFTPC*-overexpression NSCLC cells.

(A). The screening of the efficiency of si-*SOX7* sequence interference with *SOX7* gene. **(B).** The knockdown effect of si-*SOX7*-2 was assessed by qRT-RCR assay in indicated cells, the histograms were applied to quantify the relative mRNA levels. **(C).** The knockdown effect of si-*SOX7*-2 was assessed by Western blot assay in indicated cells (**a, b**), the histograms were applied to quantify the relative protein levels (**c, d**). The primary antibody against SOX7 was used in this part. β-actin was used as the internal control gene. The values were presented as the Mean ± SD, un-paired t-test. ns, no significance, **p<0.01, ***p<0.005, ****p<0.001.

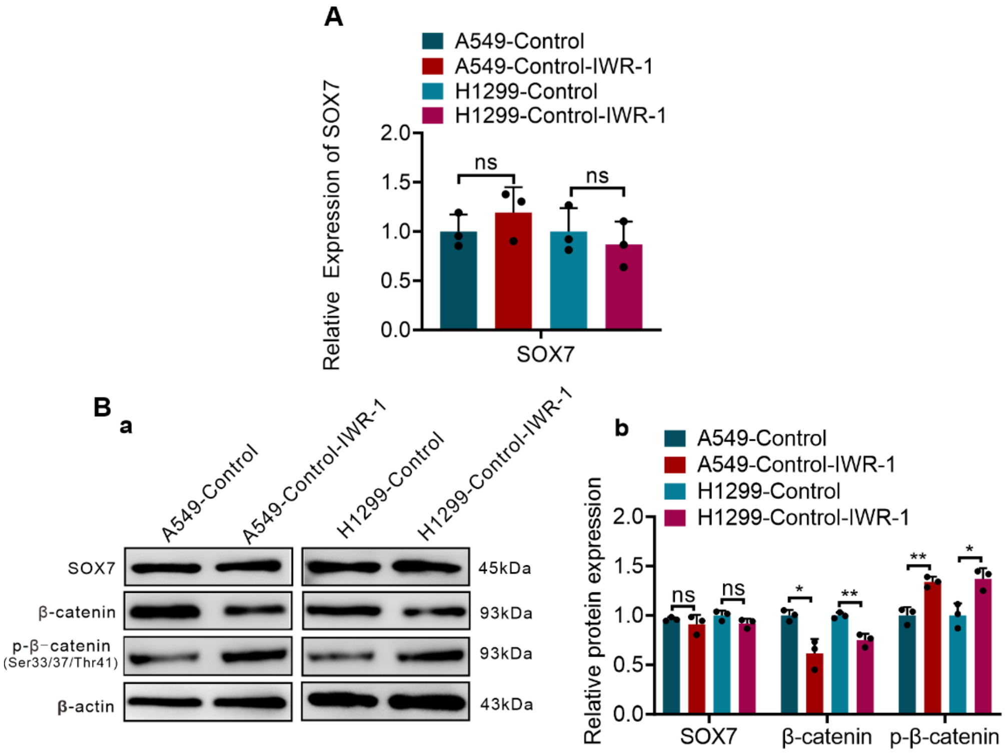

Supplementary figure 6. The detection of expression level of SOX7 in the cells after treatment with IWR-1.

(A). qRT-PCR assay was conducted on the total RNA of the indicated cells treatment with IWR-1 (0.5μM) or without IWR-1 (n=3), the relative mRNA level of *SOX7* was detected. The histogram was applied to quantify the relative mRNA level. **(B).** Western blot assay was conducted on the indicated cells treatment with IWR-1 (0.5μM) or without IWR-1 by using primary antibody against SOX7 (**a**), and the histograms were applied to quantify the relative protein level (**b**). β-actin was used as the internal control gene. All the data were presented as the Mean ± SD, un-paired t-test. ns, no significance, *$p<0.05$, **$p<0.01$. All experiments were conducted a minimum of three times independently.

2.2 Supplementary Tables

Supplementary Table S1. Clinicopathological features and relative expression of *SFTPC* in 46 LUAD tissues

No	Gender	Age	Histological	Differentiation	T stage	N stage	M stage	*SFTPC* relative expression
1	F	67	A	/	T2	N	N	0.006661
2	F	73	A	/	T1	N	N	0.02288
3	M	64	A	/	T1	N	N	0.002036
4	F	59	A	/	T1	N	N	0.0003884
5	F	68	A	/	T1	N	N	0.01199
6	M	50	A	medium-high	T2	N	N	0.06157
7	F	76	A	/	T2	N	N	0.06839
8	F	60	A	/	T1	N	N	0.001032
9	M	66	A	low	T1	N	N	0.003334
10	F	67	A	medium	T1	N	N	0.001094
11	F	64	A	medium	T3	Y	N	0.001049
12	M	78	A	low	T2	N	N	0.04349
13	F	66	A	/	T1	N	N	0.07802
14	M	35	A	low	T2	Y	N	0.02541
15	F	74	A	low	T2	N	N	1.014
16	M	66	A	medium	T1	N	N	0.01937
17	M	58	A	medium	T1	N	N	0.003813
18	M	69	A	medium	T2	N	N	0.003277
19	F	54	A	/	T1	N	N	0.001132
20	F	73	A	low	T1	N	N	0.005184
21	M	54	A	medium-low	T2	N	N	0.01253
22	F	36	A	/	T1	N	N	0.3969
23	F	46	A	high	T1	N	N	0.9977
24	F	58	A	/	T1	N	N	0.07911
25	F	54	A	/	T1	N	N	0.06999
26	F	70	A	/	T1	N	N	0.4071
27	F	69	A	/	T1	N	N	0.1368
28	M	52	A	medium-low	T2	Y	N	0.007885
29	M	71	A	medium	T2	N	N	0.1669
30	M	72	A	medium	T1	N	N	0.4747
31	F	51	A	medium-high	T1	N	N	0.1456
32	M	62	A	medium-low	T2	Y	N	0.0002294
33	M	54	A	/	T1	N	N	0.02033
34	F	60	A	medium	T1	N	N	0.21
35	F	50	A	medium	T2	N	N	0.01989
36	M	60	A	medium	T2	N	N	0.001661
37	F	68	A	/	T1	N	N	2.378
38	F	56	A	medium-high	T1	N	N	1.363
39	M	55	A	medium	T1	N	N	0.001518

40	F	57	A	/	T4	Y	N	0.08362
41	M	55	A	medium	T1	N	N	0.01553
42	M	51	A	low	T1	Y	N	0.002026
43	M	50	A	low	T4	Y	N	0.001188
44	M	67	A	/	/	/	/	0.01907
45	F	54	A	medium-low	T4	Y	N	0.005515
46	F	52	A	medium-low	T2	N	N	0.02595

Note: M, male; F, female; A, adenocarcinoma; Y, yes; N, no. Relative expression of *SFTPC* was performed by the $2^{-\Delta\Delta Ct}$ method with adjacent non-tumorous lung tissues as a calibrator. Data show the means from independent analyses. Every independent analysis by qRT-PCR was carried out immediately after the RNA extraction and reverse transcribed. ΔCt obtained from quantitative real-time PCR was subjected to paired *t*-test ($\Delta Ct = Ct_{SFTPC} - Ct_{\beta\text{-}actin}$).

Supplementary Table S2. IHC staining score criteria and outcomes

Degree	Proteins	Score
Blue	Negative (-)	0
Light brown	Weakly positive (+)	1
Brown	Positive (++)	2
Dark brown	Strongly positive (+++)	3

IHC staining

Protein	proSP-C				SOX7			
**Number**	**Adjacent non-tumor**	**Score**	**Tumor**	**Score**	**Adjacent non-tumor**	**Score**	**Tumor**	**Score**
case1	+++	3	+	1	++	2	-	0
case2	+++	3	+	1	+++	3	-	0
case3	+++	3	-	0	++	2	+	1
case4	+++	3	+	1	+++	3	+	1
case5	++	2	+	1	++	2	-	0
case6	++	2	-	0	++	2	-	0
case7	++	2	+	1	\	\	\	\
case8	+++	3	-	0	+++	3	-	0
case9	+++	3	+	1	+++	3	+	1
case10	+++	3	+	1	+++	3	+	1
case11	+++	3	+	1	\	\	\	\
case12	+++	3	+	1	+++	3	+	1
case13	+++	3	+	1	+++	3	++	2
case14	+++	3	++	2	\	\	\	\
case15	+++	3	++	2	+++	3	+	1
case16	+++	3	+	1	\	\	\	\
case17	+++	3	-	0	\	\	\	\
case18	+++	3	++	2	\	\	\	\
case19	+++	3	+	1	\	\	\	\
Case20	++	2	-	0	\	\	\	\

Supplementary Table S3. Expression of *SFTPC* in LUAD based on nodal metastasis status in TCGA database

TCGA samples	Series 1				
	**low**	**q1**	**median**	**q3**	**high**
**Normal (n=59)**	19430.765	40051.528	52442.349	60068.298	80827.802
**N0 (n=331)**	0	6.49	209.609	1061.497	4457.222
**N1 (n=96)**	0.171	4.463	75.728	581.433	2861.668
**N2 (n=74)**	0.083	2.216	53.354	738.533	3017.554
**N3(n=2)**	5.067	31.75	58.433	85.116	111.799

The data was got from TCGA database. The expression of *SFTPC* was presented as Transcript per million (TPM). q1 and q3 are the 25th and 75th percentiles, respectively.

Supplementary Table S4. The correlation of the expression of *SFTPC* and age, gender, T stage, lymph node metastasis in 45 LUAD tissues

Clinicopathological features	No. of cases (%)	*SFTPC* Relative Expression			*p*-Value
		Up-regulated n=4	Down-regulated n=39	not significant n=2	
**Age(years)**					**0.528**
≤60	24	1	22	1	
>60	21	3	17	1	
**Gender**					**0.0679**
F	26	2	22	2	
M	19	2	17	0	
**Lymph node metastasis**					**0.2343**
Y	8	0	8	0	
N	37	4	31	2	
**T stage**					**0.2185**
T1	27	0	25	2	
T2+T3+T4	18	4	14	0	

Note: M, male; F, female; Y, yes; N, no; n, numbers

Supplementary Table S5. The relationship between low expression of *SFTPC* and EMT phenotype status in LUAD tissues

EMT phenotype					
**Characteristics**	**Number**	**Epithelial (%)**	**Mesenchymal (%)**	**EMT (%)**	**Not specified (%)**
low *SFTPC* level	40	2(5.0)	21(52.5)	7(17.5)	10(25.0)

Supplementary Table S6. The expression levels of *SOX7* in LUAD tissues with low expression of *SFTPC*

SOX7 expression levels				
**Characteristics**	**Number**			
low *SFTPC* level	down-regulated (%)	up-regulated (%)	not significant (%)	Total
	27(79.4)	3(8.8)	4(11.8)	34

Supplementary Table S7. The sequences of si-RNA

si-RNA	Sequences（5'- 3'）
si-NC	UUCUCCGAACGUGUCACGUTT
	ACGUGACACGUUCGGAGAATT
si-*SOX7*-1	CCACUCCACUCCAACCUCCAATT
	UUGGAGGUUGGAGUGGAGUGGTT
si-*SOX7*-2	ACGCCGAGCUGUCGGAUGG TT
	CCAUCCGACAGCUCGGCGU TT
si-*SOX7*-3	CCACUCCACUCCAACCUCCAATT
	UUGGAGGUUGGAGUGGAGUGGTT

Supplementary Table S8. The sequences of genes for qRT-PCR

Primers	Sequences（5'- 3'）
β-actin	CTTAGTTGCGTTACACCCTTTCTTG
	ACTGCTGTCACCTTCACCGTTC
SFTPC	AGCAAAGAGGTCCTGATGGA
	ACAATCACCACGACGATGAG
SOX7	CTGCCCACACCTCCTGAAAT
	AGCTGTTGTAGTACGTGGCC
CTNNB1 (β-catenin)	TTGAAGGTTGTACCGGAGCC
	GCCACCCATCTCATGTTCCA
CDH1(E-Cadherin)	AGCAGAACTAACACACGGGG
	ATACCGGGGGACACTCATGA
CDH2(N-Cadherin)	TGCCGGTACCATGTTGACAA
	CACTGGCAAACCTTCACACG
Snai2(Slug)	CATCTTTGGGGCGAGTGAGT
	ACACAGCAGCCAGATTCCTC

Reference

1. Yuan Y, Liao H, Pu Q, Ke X, Hu X, Ma Y, et al. miR-410 induces both epithelial-mesenchymal transition and radioresistance through activation of the PI3K/mTOR pathway in non-small cell lung cancer. Signal transduction and targeted therapy. 2020;5(1):85.
2. Zhu Y, Pu Q, Zhang Q, Liu Y, Ma Y, Yuan Y, et al. Selenium-binding protein 1 inhibits malignant progression and induces apoptosis via distinct mechanisms in non-small cell lung cancer. Cancer Med. 2023;12(16):17149-70.
